# Strategies for Delivery of siRNAs to Ovarian Cancer Cells

**DOI:** 10.3390/pharmaceutics11100547

**Published:** 2019-10-22

**Authors:** Rossella Farra, Matea Maruna, Francesca Perrone, Mario Grassi, Fabio Benedetti, Marianna Maddaloni, Maguie El Boustani, Salvo Parisi, Flavio Rizzolio, Giancarlo Forte, Fabrizio Zanconati, Maja Cemazar, Urska Kamensek, Barbara Dapas, Gabriele Grassi

**Affiliations:** 1 Department of Life Sciences, Cattinara University Hospital, Trieste University, Strada di Fiume 447, I-34149 Trieste, Italy; rfarra@units.it (R.F.); mateamaruna93@gmail.com (M.M.); francesca.perrone@phd.units.it (F.P.); marianna.maddaloni@libero.it (M.M.); ggrassi@units.it (G.G.); 2 Department of Engineering and Architecture, University of Trieste, Via Valerio 6/A, I-34127 Trieste, Italy; mario.grassi@dia.units.it; 3 Dipartimento di Scienze Chimiche e Farmaceutiche, Università degli Studi di Trieste, I-34127 Trieste, Italy; benedett@units.it; 4 Pathology Unit, IRCCS CRO Aviano-National Cancer Institute, I-33081 Aviano, Italy; elboustanymaguie@gmail.com (M.E.B.); kika.grassi.0671@gmail.com (S.P.); frizzolio1@gmail.com (F.R.); 5 Doctoral School in Molecular Biomedicine, University of Trieste, I-34127 Trieste, Italy; 6 Department of Molecular Sciences and Nanosystems, Ca’ Foscari University of Venice, I-30123 Venezia-Mestre, Italy; 7 International Clinical Research Center (ICRC), St Anne’s University Hospital, CZ-65691 Brno, Czech Republic; giancarlo.forte@fnusa.cz; 8 Department of Medical, Surgical and Health Sciences, University of Trieste, Cattinara Hospital, Strada di Fiume, 447, I-34149 Trieste, Italy; fabrizio.zanconati@asuits.sanita.fvg.it; 9 Department of Experimental Oncology, Institute of Oncology Ljubljana, Zaloska 2, SI-1000 Ljubljana, Slovenia; MCemazar@onko-i.si; 10 Faculty of Health Sciences, University of Primorska, Polje 42, SI-6310 Izola, Slovenia; UKamensek@onko-i.si

**Keywords:** ovarian cancer, siRNA, polymer, lipid, delivery

## Abstract

The unmet need for novel therapeutic options for ovarian cancer (OC) deserves further investigation. Among the different novel drugs, small interfering RNAs (siRNAs) are particularly attractive because of their specificity of action and efficacy, as documented in many experimental setups. However, the fragility of these molecules in the biological environment necessitates the use of delivery materials able to protect them and possibly target them to the cancer cells. Among the different delivery materials, those based on polymers and lipids are considered very interesting because of their biocompatibility and ability to carry/deliver siRNAs. Despite these features, polymers and lipids need to be engineered to optimize their delivery properties for OC. In this review, we concentrated on the description of the therapeutic potential of siRNAs and polymer-/lipid-based delivery systems for OC. After a brief description of OC and siRNA features, we summarized the strategies employed to minimize siRNA delivery problems, the targeting strategies to OC, and the preclinical models available. Finally, we discussed the most interesting works published in the last three years about polymer-/lipid-based materials for siRNA delivery.

## 1. Introduction

Effective therapeutic approaches are lacking for ovarian carcinoma (OC), the most lethal gynecological neoplasm. Thus, the development of novel strategies is of the utmost urgency. In this review, we focused our attention on the use of small interfering RNAs (siRNAs), short double stranded RNA molecules with the ability to downregulate the expression of virtually any gene causing disease in humans [1,2]. However, due to their fragile nature in the biological environment, siRNAs, as many other nucleic acid based molecules [3,4,5], need to be complexed with adequate delivery materials. Among these, we focused herein on polymers and lipids, which show high biocompatibility and the ability to deliver siRNAs. Other reviews have covered additional delivery systems [6,7,8,9].

For the development of effective delivery strategies and novel therapeutic drugs for OC, different aspects should be considered. The first is the biological/anatomical features of OC. For this reason, we have dedicated specific sections to this topic (Section 1.1, Section 1.2 and Section 1.3). The second aspect is related to the specific nature of siRNAs, as well as the problems of their delivery in general and in particular to OC cells (Section 2.1 and Section 2.2). The third element involves the features of polymer- and lipid-based delivery materials, the general characteristics of which have been described (Section 3.1 and Section 3.2). A fourth aspect relates to the possibility of generating siRNA delivery complexes able to target OC cells (Section 4.1) and/or OC-specific gene products (Section 4.2) to minimize side effects and improve effectiveness. The fifth aspect relates to the employment of models adequate to produce data with potential value in humans (Section 5.1, Section 5.2 and Section 5.3). Finally, we have presented the most recent works (from the last three years, Section 6) related to the delivery of siRNAs, also making methodological considerations (Section 7).

### 1.1. Ovarian Carcinoma

Ovarian carcinoma (OC), the seventh most common cause of death among women in industrialized countries, is the most lethal gynecological neoplasm [10]. In women under 40 years of age, OC is rare; however, the risk increases above age 40, peaking in the late 70s [10]. Annually, 240,000 new diagnoses are registered worldwide, with a higher incidence in the Caucasian population in the countries of northwestern Europe and in the USA compared to Asian, African, and South American countries [11]. As OC does not have a specific symptomatology in the initial phases, about 70% of OCs are diagnosed in advanced stages when there are already abdominal metastases [12]. For the advanced forms, the combined association of surgical removal of cancer and chemotherapy has a five-year survival rate of 25% [13]. Moreover, disease recurrence within two years of diagnosis is frequent [14], and when this occurs, OC very frequently displays a high resistance to chemotherapy. Together, these factors explain why the five-year survival in OC has remained almost stable (38% 1990–1994; 41% 2000–2004) in recent years.

### 1.2. Classification and Biology of OCs

**Figure 1:**
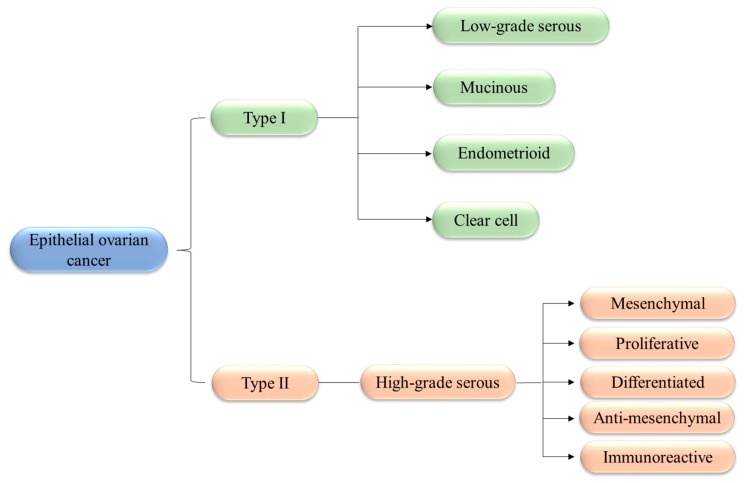
Histological classification of epithelial ovarian carcinoma.

From a histological point of view, OCs can be divided into three subtypes based on the cells of origin. Epithelial tumors, representing more than 90% of OCs, originate from the epithelial cells that line the surface of the ovaries or the fimbriae. Germ cell tumors, accounting for just 5% of OCs, originate from germ cells and are almost exclusively found in juvenile populations. Finally, stromal tumors, constituting about 4% of OCs, originate from the gonadal stroma, which supports ovary tissue. OCs of epithelial origin have been further subdivided into (Figure 1): type I tumors, which contain well-differentiated cells (such as low grade serous carcinoma and endometrioid carcinoma) and are characterized by low malignity; type II tumors, which are rather aggressive and arise directly from the epithelial tissue of the organ without going through a precancerous phase (typically represented by the high-grade serous ovarian carcinomas—HGSOCs) [15]. Gene expression profiling has revealed the existence of five molecular subtypes of HGSOCs: mesenchymal; proliferative, with the worst overall survival; differentiated, with an intermediate prognosis; anti-mesenchymal; and immunoreactive, with a better prognosis [16,17,18].

**Figure 2:**
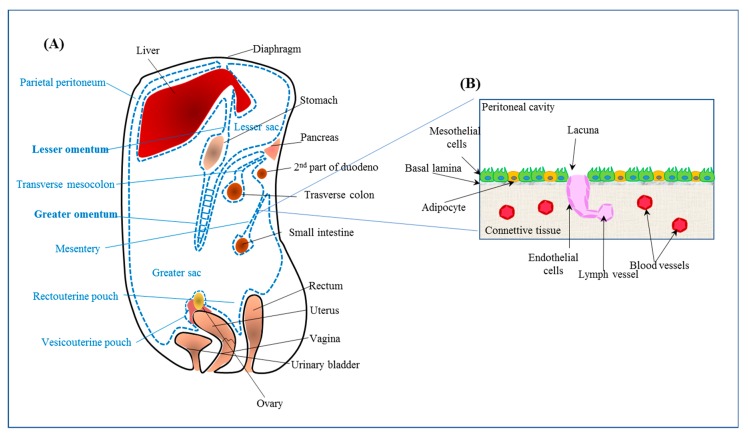
(**A**) Anatomical distribution of the peritoneum; the blue dotted line indicates the peritoneum. (**B**) Cellular structure of the peritoneum.

HGSOC is the most common (70%) and most aggressive OC histotype [19]. The survival for early-stage and advanced-stage patients 10 years from diagnosis is 55%, and 15%, respectively [20]. In contrast to many other tumors, HGSOC does not require the blood or lymph to metastasize, as it can spread by direct contact to the neighboring organs within the peritoneal cavity or through cell exfoliation from the primary tumor [19]. Although, in principle, every organ within the peritoneal cavity may be involved in tumor dissemination, the involvement of a part of the neighboring peritoneum named omentum is the most frequent. The peritoneum is a large serous membrane extending from the stomach and covering the intestines (Figure 2), which contains energy-dense adipocytes. It has been proposed that HGSOC predilection for the omentum is due to cancer cell metabolism, which requires fatty-acid β-oxidation [21].

### 1.3. Current Therapies

The primary intervention for HGSOC is based on the use of surgery with the aim to remove the macroscopic lesions including metastasis [22], which are, as mentioned above, predominantly localized in the omentum. Notably, because of the specific biology of HGSOC, metastasis removal can improve overall survival. After surgery, the vast majority of patients undergo chemotherapy, which is the same for any type of OC [23]. Current chemotherapy is based on the use of the combined systemic administration of cisplatin–paclitaxel (PTX) [24]. More recently, intra-peritoneal (IP) administration of drugs has been proposed. As HGSOC is mostly confined to the peritoneal cavity, the IP route enables a higher local concentration [24]. Notably, a recent meta-analysis showed that IP administration significantly improved progression-free survival in advanced HGSOC patients compared to systemic administration [25]. However, significant toxicity is still an issue to be solved.

About 70% of patients treated for HGSOC respond to the first-line, PTX-based chemotherapy [26]. However, about 80% of these relapse soon or later [22]. Within relapsing patients, about 50% are still responsive to PTX, even though persistent side effects from previous treatment may become problematic. For PTX-resistant patients, alternative options are limited and poorly effective [22]. Novel therapeutic approaches include the use of poly (ADP-Ribose) polymerase (PARP) inhibitors. PARP mediates base excision repair of single-strand breaks to resolve spontaneous DNA damage [27]. Thus, PARP inhibition can expose the cancer cell to increased DNA damage, eventually leading to cell death.

## 2. Small Interfering RNAs

**Figure 3:**
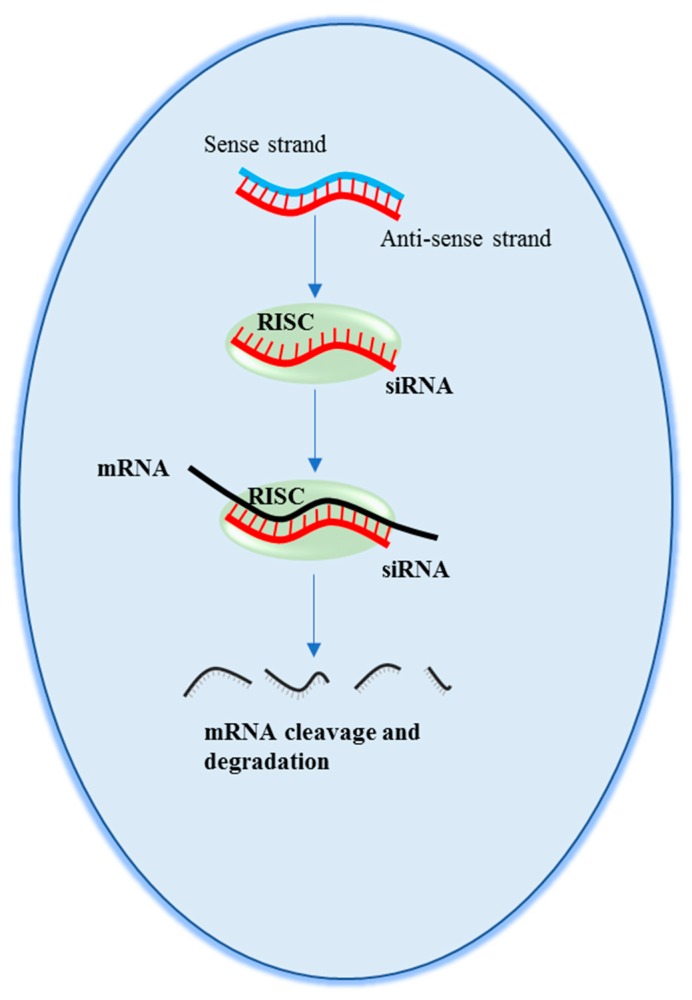
Short interfering RNA (siRNA) mechanism of action.

Small interfering RNAs (siRNAs) were first described about three decades ago in plants [28], and then in *Caenorhabtidis elegans* [29]. Their therapeutic potential was first reported in 2001 [30], when it was shown that a short, double-stranded RNA duplex (about 21 nucleotide long) can induce target mRNA degradation, thus suppressing gene expression in human cells. The mechanism of action (Figure 3) begins with the uptake of one of the two RNA filaments (antisense strand) of the siRNA by a cellular protein complex termed RISC (RNA-induced silencing complex). While the other filament (sense strand) is discarded, the antisense strand drives RISC to a target RNA via a perfect sequence complementarity. This, in turn, allows RISC to induce the degradation of the target RNA, leading to gene expression inhibition. It is possible to design siRNAs targeted against virtually any deleterious (m)RNA, including viral RNA [31,32]. Moreover, their chemical synthesis is neither complex not particularly expensive. The above characteristics make the therapeutic applicability of siRNA potentially very broad [2,33,34,35,36].

### 2.1. Delivery Barriers for Systemic Administration

**Figure 4:**
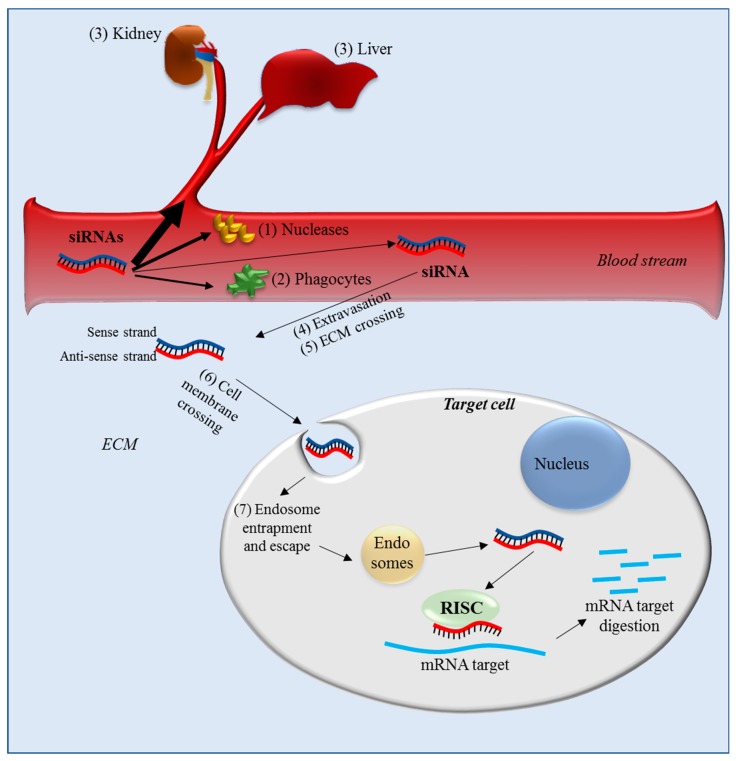
Obstacles for systemic siRNA delivery.

Made of RNA, siRNAs are characterized by a negative electric charge and have poor stability in the biological environment. Thus, siRNAs need to be embedded into specific carriers for protection and to efficiently reach the target cells. It should be considered that for systemic administration, as could be the case in treating ovarian cancer, siRNAs encounter a number of obstacles that can dramatically reduce the possibility of their reaching the target cancer cells. Once in the blood stream, siRNA can: (1) be degraded by blood nucleases, (2) be eliminated by the phagocytic system, (3) be cleared from blood via kidney filtration and/or sequestered by the liver [37] and activate the innate immune response [38] (Figure 4). Once the siRNAs reach the target tissue, additional obstacles remain, like (4) crossing the vessel wall (extravasation), (5) the migration through the extracellular matrix (ECM) and then (6) crossing the cellular membrane (Figure 4). This last step is particularly inefficient for naked siRNA as their global negative charge, derived from the phosphate groups of their backbone, induces the repulsion of siRNA from the negatively charged molecules present on the outer side of the cell membrane. Moreover, the hydrophilic nature of siRNA substantially prevents its crossing through the hydrophobic inner layer of the cell membrane. Only a reduced amount of siRNA can be internalized via endocytosis. Finally, once into the target cell, siRNAs can be entrapped in endosomes (7), an event that can further reduce the amount of siRNA able to reach the target.

### 2.2. Delivery Barriers for IP Administration

An emerging drug administration strategy for ovarian cancer is the IP route. Thus, it might feasible to develop an IP delivery system for siRNAs as well. Considering that ovarian cancer metastasis very frequently targets the peritoneum/omentum, knowledge of the biology of this serous membrane is crucial to designing optimal siRNA delivery systems. The surface of the peritoneum contains a single layer of mesothelial cells (Figure 2) with a cuboidal shape and adipocytes (reviewed as reported by Sarfarazi et al [39]). Tight junctions and plasmalemma interdigitations connect mesothelial cells. Underneath the mesothelial cells there is basal lamina, which separates the cells from the connective tissue layer. This last layer, composed of collagen fibers, elastin, and fibroblasts, is characterized by the presence of irregular oval openings with diameters ranging from 3 to 15 μm, distributed irregularly in the layer. Dispersed on the peritoneum surface are lymphatic lacunae, lined by endothelial cells. The lacunae are in connection with lymphatic capillaries. In lacunae, MS and endothelial cells are close to each other and share a common basal lamina. The diameter of lacuna aperture is 1.8–6 μm in most animal species, and 3.2–3.6 μm in humans. Blood vessels are abundantly scattered in the connective tissue layer [39].

Following IP administration, delivery systems with a size similar to the diameter of the lymphatic lacuna are preferentially drained from the peritoneum via lacunae. Smaller delivery systems (<10 nm diameter) are mostly cleared via the blood, as they can more efficiently penetrate blood capillary walls. Moreover, the higher flow rate (100–500-fold) in blood vessels compared to lymphatic capillaries favors the diffusion of small molecules into the blood [40]. Thus, the retention of delivery systems in the peritoneal cavity can be modulated by their size. Delivery systems larger than the diameter of the aperture of the lymphatic lacunae will persist longer in the peritoneal cavity; those inferior in size will be predominantly cleared via the lacunar route, while the very small ones (nm range) will be mostly drained by blood vessels. Peritoneal persistence can be also improved by using positively charged delivery systems that can easily interact with the negatively charged membrane of MS [41]. Prolonged peritoneal persistence can present an advantage, as ovarian cancer metastasis typically colonizes the mesothelial cell layer without affecting the basal lamina [42]. Obviously, tumor cell membrane crossing, the presence of cytosolic nucleases, and endosome entrapment still remain obstacles for siRNA action in the case of IP administration.

## 3. Strategies to Optimize siRNA Delivery

**Figure 5:**
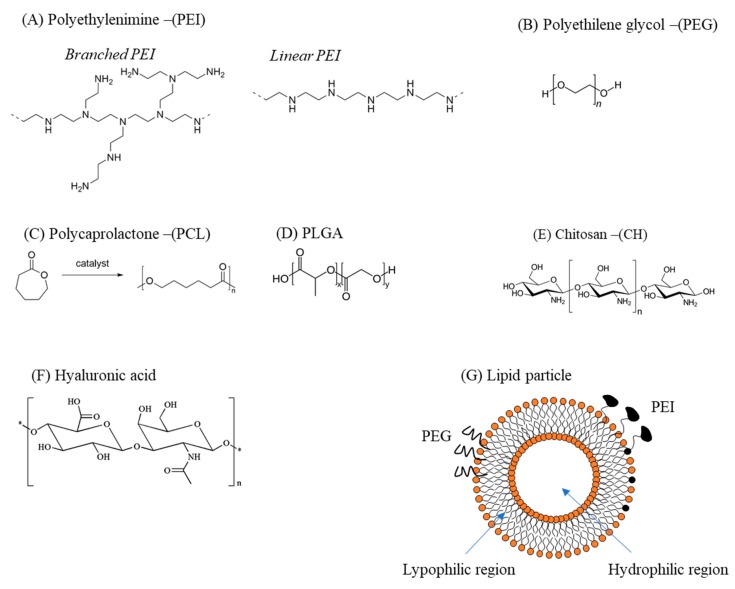
Structure of polymer- and lipid-based delivery materials, (**A**) polyethylenimine (PEI), (**B**) polyethylene glycol (PEG), (**C**) polycaprolactone (PCL), (**D**) poly(lactic-*co*-glycolic acid) (PLGA), (**E**) chitosan (CH), (**F**) hyaluronic acid (HA), and (**G**) lipid particles.

The fragile nature of siRNAs in the cellular and extracellular environment prevents their use as naked molecules. To improve their stability and thus effectiveness, it is possible to chemically modify their structure to make them more resistant to degradation [2,33]. An additional and complementary strategy consists of the combination of siRNAs with delivery vectors able to preserve their integrity and drive them to the target cell [2,33]. Additionally, electroporation can be also used [43]. Often, but not always, the two strategies are combined together. However, as chemical modifications can impair siRNA effectiveness, their employment should be carefully considered. Here, we focus on a brief description of the most commonly used delivery materials, i.e., those based on polymers and lipids (Figure 5).

### 3.1. Polymer-Based Delivery Vectors

Polymers have been frequently used as siRNA delivery systems due to their versatility, low cost of production/isolation, and biocompatibility. The most commonly used polymers for siRNA delivery are polyethylenimine (PEI), polyethylenglycole (PEG), polycaprolactone (PCL), poly(lactic-*co*-glycolic acid) (PLGA), chitosan (CH), and hyaluronic acid (HA). These polymers are frequently combined with each other and/or with other molecules to create delivery particles with optimized delivery features. Regardless of the chemical nature of the polymer, they need to have positively charged resides to bind the negatively charged siRNAs via electrostatic interactions. Moreover, the resulting polymer–siRNA complex should have an appropriate surface charge: excessively strong cationic complexes can lead to nonspecific cellular uptake [44,45,46] and nonspecific membrane disruption. On the other hand, anionic particles that are too strong can have problems interacting with the negatively charged cellular membrane, thus resulting in inefficient intracellular delivery of siRNA.

Repeated units composed of amine groups and two carbon aliphatic CH_2_–CH_2_ spacers (Figure 5A) form the structure of PEI. It can exist in both linear and branched forms: in the first form, it contains all secondary amines, while in the second form it contains primary, secondary, and tertiary amino groups. The amino groups confer a cationic nature to PEI, suitable for binding siRNAs. High-molecular-weight PEI displays a superior ability to carry and deliver siRNAs compared to low-molecular-weight [47]. However, high-molecular-weight PEI is more cytotoxic than low-molecular-weight PEI, a problem that can be attenuated by the addition of hydrophilic and hydrophobic moieties or cell-/tissue-specific ligands [48]. Finally, due to its ability to promote the “proton sponge effect”, PEI favors siRNA endosomal escape [49].

The structure of PEG is represented by the formula H−(O−CH_2_−CH_2_)*_n_*−OH (Figure 5B). Being substantially non-toxic [50] and soluble in water, PEG can be used in many biological applications. PEG can reduce the toxicity of other delivery materials and, because of this property, it is often added (PEGylation) to various delivery particles [51]. Moreover, it can be also used as a linker for specific ligands on the surface of delivery particles [52].

PCL is obtained by the ring opening polymerization of the cyclic monomer ε-caprolactone (Figure 5C) [53]. It is biodegradable and, in the field of drug delivery, it is typically used to tune the physical, chemical, and mechanical properties of different materials when co-polymerized with polymers such as PEG. Its main limitation in siRNA delivery is the lack of cationic groups able to interact with the negative siRNA backbone [54]. Thus, PCL is often combined with PEI and/or PEG.

PLGA is a copolymer of poly(d, l-lactic acid; LA) and poly(glycolic acid; GA) linked together through ester linkages [54] (Figure 5D). PLGA, biodegradable into its original monomers LA and GA, has been approved for human use by the US Food and Drug administration. PLGA gained attention as an siRNA delivery material due to its favorable safety profile and sustained-release characteristics. However, one of the weaknesses of PLGA is the modest siRNA loading capacity due to the repulsion between the anionic acid groups in PLGA and the negative phosphate backbone of siRNA. To minimize this problem, PLGA is often combined with cationic molecules such as dioleyltrimethylammoniumpropane (DOTAP) or PEI.

It is possible to commercially obtain CH by deacetylation of chitin, a substance contained in the exoskeletons of crustaceans and the cell walls of fungi, [55] (Figure 5E). Chitosan is a linear polysaccharide with a carbohydrate backbone with two types of repeating residues, 2-amino-2-deoxy-glucose (glucosamine) and 2-*N*-acetyl-2-deoxy-glucose (*N*-glucosamine), linked by a (1-4)-β-glycosidic linkage. The amino groups confer to CH a positive charge, which allows binding with the negatively charged phosphate groups of siRNAs. CH conjugation with PEI and PEG can bypass the problems of low solubility and delivery effectiveness. Alternatively, it is possible to tune the degree of acetylation and modify the molecular weight [56].

HA, a polymer of natural origin, is a linear polysaccharide formed by glucuronic acid and N-acetylglucosamine units linked via alternating –1,4 and –1,3 glycosidic bonds (Figure 5F). HA is biocompatible, biodegradable, and characterized by low toxicity. In the field of siRNA delivery, it is often used in combination with other cationic polymers. Indeed, its anionic nature attenuates positive charges, thus resulting in reduced toxicity and improved stability of the complex [57]. Finally, the presence of hyaluronic acid receptors (CD44, see Section 4) in many healthy and diseased human (cancer) tissues makes HA particularly suited as a targeting agent [58].

### 3.2. Lipid-Based Delivery Vectors

Liposomes are spherical vesicles formed by concentric lipid bilayers (Figure 5G) with a hydrophilic region localized in the core [59,60]. Because of this structure, liposomes can entrap hydrophilic molecules in the water compartment and hydrophobic molecules in the lipid layers. Frequently, liposomes are made up of neutral (cholesterol; Chol) or zwitterionic (dioleoylphosphatidylethanolamine; DOPE) lipids, giving origin at the physiological pH to neutral liposomes [60]. While neutral liposomes have good biocompatibility and favorable pharmacokinetics, they display poor interactions with negatively charged siRNA. An alternative to the use of neutral lipids is the employment of positively charged lipids, such as 3β[*N*-(*N*’,*N*’-dimethylaminoethane)-carbamoyl]DC-cholesterol, 1,2-dioleoyl-3-trimethylammonium propane (DOTMA) [60]. Cationic liposomes are composed by positively charged lipids within a matrix of neutral lipids, required for cellular internalization. Cationic liposomes allow easy and effective loading of negative molecules such as siRNA. Moreover, they facilitate the endosomal escape of siRNA, promoting the fusion between liposomal and endosomal bi-layers. Despite being suitable for siRNA delivery to the cells, cationic liposomes have some drawbacks [59]. For example, they are more toxic than neutral liposomes. They also have a low “fusogenicity” attitude, i.e., they are less effective in interacting with cell membranes to allow cell uptake. To attenuate this limitation, cationic lipids are often combined with non-cationic lipids such as DOPE, which display enhanced fusogenic activity. Another limitation of cationic liposomes is represented by the fact that, when applied to the blood stream, they are cleared by the reticulo-endothelial system. Thus, their effectiveness in siRNA delivery can be significantly diminished. To minimize this problem, cationic liposomes are often equipped with shielding molecules on their surface such as a hydrophilic polymer like PEG. However, once they reach the target cell, the PEG has to be removed to allow efficient siRNA delivery and to permit efficient endosome escape.

## 4. Targeting OC: Delivery Systems and siRNA Targets

The ideal therapeutic approach to OC should be able to selectively deliver the drug (siRNA in our case) to tumor cells, leaving the normal tissues as untouched as possible. This goal can be approached with two complementary strategies: the first is to equip the siRNA delivery system with molecules able to specifically target tumor cells, and the second is to use siRNAs against OC-specific oncogenes. The combination of these two strategies should give the highest specificity level possible.

### 4.1. OC-Targeting Molecules

So far, different antigens present on the surface of OC cells have been considered suitable for OC targeting (Table 1).

Epidermal growth factor receptor (EGFR) is highly expressed in OC [61], as well as in other tumor cells. An extracellular ligand-binding domain, a transmembrane domain, and a cytoplasmic domain containing the tyrosine kinase region [62] characterize this surface antigen. Following ligand binding to EGFR, receptor auto-transphosphorylation in the tyrosine kinase region occurs, triggering a series of signaling events. These, in turn, promote cell proliferation, apoptosis inhibition, cell invasion, and stimulation of neovascularization [63]. Because of its pro-proliferative effects, EGFR has also been considered as a drug target [64].

Erythropoietin-producing hepatocellular receptor A2 (EphA2) belongs to a large family of receptor tyrosine kinases mainly involved in the regulation of cell proliferation and migration [65]. The extracellular domain contains a ligand-binding domain, a Sushi domain, an epidermal growth factor (EGF)-like domain, and two fibronectin type-III repeats. A transmembrane domain and a tyrosine kinase domain follow the extracellular domain. EphA2 is overexpressed in ovarian cancer [66] as well as in various other solid tumors, such as breast, prostate, pancreas, glioblastoma, neck, renal, lung, melanoma, bladder, gastric esophageal, colorectal, and cervical cancers. In OC, the N-terminals of EphA2 are processed by membrane-type 1 matrix metalloproteinase, triggering ligand-independent signal activation, which in turn promotes cell motility, invasion, and metastasis [66].

The folic acid (FA) receptor (FR) is a 38 kDa glycosyl-phosphatidylinositol membrane-anchored glycoprotein [67]. Under normal conditions, FR is present only in some polarized epithelia and it is localized to the apical/luminal cell surface. In tumor cells of epithelial origin, it is often overexpressed and it loses its polarized nature, covering the entire cell surface. FR acts as a transporter of folate into the cells. The fact that folates are essential for the biosynthesis of purines and thymidine, necessary for DNA synthesis, methylation, and repair, explains why actively growing tumor cells need higher FR compared to normal cells. Notably, FR overexpression is present in more than 80% of HGSOCs [68]. Moreover, increased expression level of FR correlates with OC stage, poor response to chemotherapy, and worse survival.

In addition to the above-mentioned OC-targeting antigens, other antigens associated with ovarian cancer stem cells (OCSCs) are gaining great interest. The discovery of quiescent OCSCs with the ability to escape chemotherapy and to regenerate OCs has directed attention towards the possibility of specifically targeting OCSCs [69]. CD44 is a surface transmembrane glycoprotein that acts as a receptor for different molecules, including hyaluronic acid (HA). CD44 influences the expression levels of genes related to cellular differentiation and cell–matrix adhesion. It is one of the most common OCSC surface markers, used to identify this kind of cell. Despite this, CD44 does not seem to have relevant prognostic value in OC [70]. Monoclonal antibodies and siRNAs [71] directed against CD44 have already been tested. Another OCSC-related antigen is CD133, a glycosylated transmembrane protein with prognostic value in OC. CD133 modulates several pathways involved in the control of cancer stemness and metastasis. Notably, even though CD133-positive cells represent a minority of OC cells, its toxin-mediated targeting effectively downregulated OC growth in in vitro and in vivo models [72]. Further potential targeting antigens for OC are CD117 and CD24. CD117, also known as c-kit, is a receptor tyrosine kinase devoted to the promotion of cell survival, metabolism, and differentiation. In OC, poor disease-free survival is directly associated with CD117 levels [73]. While CD117 targeting in vitro successfully downregulated OC cell survival [74], only modest effects were observed in a phase II clinical trial [75]. Finally, another potential targeting antigen is represented by CD24, highly expressed in many cancers with a percent positivity in OC of about 70% [76].

### 4.2. siRNA Targets in OC

Due to their mechanism of action, siRNAs are particularly suited to targeting gene products of which the exuberant expression is responsible for increased cell growth, cell survival, migration, angiogenesis, and drug resistance (Table 2, Table 3 and Table 4). Notably, a single gene product often controls many of the above cellular phenotypes.

#### 4.2.1. Molecular Targets Implicated in Cell Growth/Migration

One of the most targeted gene products in OC is EGFR, as it promotes cell growth, apoptosis inhibition, cell invasion, and neovascularization (Table 2). This gene product is also particularly attractive as it represents a targetable surface antigen for an siRNA delivery system [64] (see Section 4.1). The expression of the *Metastasis associated in colon cancer 1* (*MACC1*) is often upregulated in many cancers. Its targeting by short hairpin shRNA in OC cells resulted in the downregulation of cell migration and proliferation [77]. *Metastasis-associated gene 1* (*MTA1*) is another gene product relevant for invasion and metastasis in OC. Notably, its siRNA-mediated down-regulation not only reduced cell migration, it also promoted cell anoikis [78]. This is a form of apoptosis occurring in cells detached from the extracellular matrix. Anoikis resistance is relevant for the survival of OC cells in ascites. The *Wilms tumor gene* (*WT1*) is overexpressed in OC but not in normal ovarian tissue; thus, it is of particular interest in terms of the specificity of siRNA effects (limited to OC cells). *WT1* targeting resulted in OC cell arrest in the G0–G1 phase of the cell cycle and promoted cell apoptosis [79]. Rac1 is a well characterized member of the Rho family that interacts with effector molecules and regulates cytoskeleton organization and membrane trafficking [80]. Rac-1 is implicated in OC cell survival, tumor angiogenesis, resistance to therapeutics, and contribution to extraperitoneal dissemination [81]. Deregulation of *Polo-like kinase 1 (Plk1)* was shown to be responsible for mitotic defects by affecting cell cycle checkpoints, thus resulting in aneuploidy and tumorigenesis [82]. Overexpression of *Plk1* has been observed in many cancerous tissues, including OC [83], and was shown to correlate with tumor stage and grade and poor patient prognosis. *Notch1, Claudin3 (CLDN3), Nin one binding protein (NOB1p)*, and *Cyclooxigenase-2 (COX-2)* (reviewed in [61]), represent other suitable targets related to OC growth and migration.

In addition to the above-discussed siRNA targets, there are others, such as *E2 promoter binding factor 1* (*E2F1*) and *peptidylprolyl cis–trans isomerase, NIMA-interacting 1* (*PIN1*), which we believe might merit the attention of future experimentation. *E2F1* belongs to a family of transcription factors with the ability to activate or repress the expression of genes promoting cell proliferation. *E2F1* in particular mostly triggers the transcription of pro-proliferative genes, thus favoring cell growth. In OC, its level directly correlates to unfavorable disease-free and overall survival, particularly in HGSOC (reviewed in Reference [84]). Moreover, recent evidence connects *E2F1* over-expression with reduced drug sensitivity [85]. Together, these findings make *E2F1* an attractive novel molecular target in OC. However, as *E2F1* also plays a relevant role in non-tumor cells, the use of an OC-targeted delivery system would be desirable. Among *E2F1* transcription targets, there is *PIN1* [86,87]. It accelerates the conversion of cis and trans isomers, thus inducing conformational changes able to regulate the functions of its substrates, such as *p53, p73, p27^Kip1^, p21^waf1/cip1^*, and *cyclin D1*, all proteins involved in the control of cell proliferation. The net result is the activation of oncogenes and inactivation of tumor suppressor genes in cancer cells. In OC (HGSOC), *PIN1* is overexpressed and when knocked down or chemically inhibited, OC cell death is induced [88,89]. The fact that *E2F1* regulates *PIN1* transcription and that both genes are involved in OC make this molecular circuit an interesting target for novel therapeutic approaches in OC.

#### 4.2.2. *Molecular Targets Implicated in Angiogenesis*

Cancer neo-angiogenesis is necessary to support the increased demand for oxygen and nutrients of the growing tumor cells. Thus, the targeting of one or more of the elements of the angiogenic pathway has the potential to negatively regulate tumor cell growth. In several tumors, including OC, different components of the angiogenic pathway have been targeted with different therapeutic molecules [61]. Among these, the vascular endothelial growth factors (VEGFs) and their tyrosine kinase receptors (VEGFR-1/Ftl-1, VEGFR-2, VEGFR-3/Ftl-4) have often been considered (Table 3). Some studies have also considered the targeting of *plexin domain containing 1 (PLXDC1*), previously known as TEM7, which is overexpressed in endothelial cells of all four tumor types [90]. *PLXDC1* promotes endothelial cell migration and invasion.

#### 4.2.3. Molecular Targets Implicated in Drug Resistance

About 80% of OC patients, who respond to the first-line PTX chemotherapy relapse sooner or later [22]. Thus, the development of drug resistance in OC is a major problem. To try to circumvent this aspect, gene products responsible for multidrug resistance have been considered as possible targets for novel therapeutic approaches (Table 4). *Multidrug resistance gene 1 (MDR1)* is a membrane-bound P-glycoprotein overexpressed in chemo-resistant OC cells [91]. MDR1 favors drug efflux from the cells, thus resulting in a shortening of drug permanence into the cell. This, in turn, leads to the reduced therapeutic effects of the drug. Another gene product related to drug resistance is *survivin (SVV)*. As member of the *Inhibitor of apoptosis protein (IAP)* family [92], *SVV* induces chemotherapy and radiotherapy resistance in OC [93] through the inhibition of apoptosis. As this protein is overexpressed in many tumors compared to their normal tissue counterparts, it represents an attractive target for siRNA tumor-specific effects. Another target able to induce chemo-resistance is the *Focal adhesion kinase (FAK)*. This is a non-receptor tyrosine kinase of which the overexpression is present in 68% of epithelial OCs [94]. Moreover, *FAK* levels significantly correlate with shorter overall patient survival. Not only is *FAK* involved in the regulation of OC cell migration, invasion, adhesion, proliferation, and survival, it has recently been discovered that it promotes PTX resistance in OC as well as in other cancers [94]. Drug resistance is triggered via the promotion of *MDR-1* activity. Finally, *B-cell lymphoma 2 (BCL-2)* is the founding member of the *BCL-2* family of apoptosis regulatory proteins. In particular, *BCL-2* has anti-apoptotic function, and, recently, its involvement in the induction of chemo-resistance in OC has been described. Indeed, its targeting resulted in enhanced cisplatin-induced apoptosis in OC spheroids [95].

## 5. Experimental Models of OC

The development of effective anti OC siRNA and delivery strategies depends on the employment of adequate models of the disease. Available models include cellular and animal models. Recently, conglomerates of cells cultured in 3D and defined spheroids have been proposed as a useful model. Finally, mathematical modeling is gaining interest as a way to better understand the biological phenomena in siRNA effects and delivery. Herein, we have briefly summarized the features of the available models, highlighting their advantages and disadvantages.

### 5.1. Cellular Model

The ideal cellular model should be able to replicate the main phenotypic and genetic characteristics of OC cells. As HGSOC is the most problematic form of OC, the cellular models should ideally resemble the features of this form of OC. In HGSOC, copy number alterations (CNAs) are rather common; more frequent CNAs involve genes such as *MYC*, *MECOM*, *CCNE1,* and *KRAS* [96]. Additionally, some gene mutations are also common: among these, *TP53* mutations are present in 95% of the cells isolated from patients [97] and *BRCA1/BRCA2* have a frequency of about 10%. Notably, low-grade serous carcinoma has almost normal gene copy numbers and wild-type *TP53* [98]. Based on these and other molecular features, a classification of the cellular models that best resemble HGSOC has been proposed [97]. The most commonly used model cell lines, represented by SKOV3, A2780, OVCAR3, CAOV3, and IGROV1 [97], do not properly resemble HGSOC. OVCAR3 and CAOV3 possess *TP53* mutations like HGSOC, but have low CNAs compared to HGSOC. SKOV3 and A2780 do not have *TP53* mutations, but instead do have mutations frequently found in other histological subtypes, such as *ARID1A*, *BRAF*, *PIK3CA*, and *PTEN* mutations. IGROV1, characterized by few CNAs and many mutations not present in HGSOC, resembles the molecular features of the endometrioid carcinoma. The HeyA8 cell line has fewer *CNAs* than HGSOC and do not have mutated *TP53*, *BRCA1*, and *BRCA2*. In contrast, a cellular model that reasonably well resembles the molecular features of HGSOC is the KURAMOCHI cells. They have similar CNAs and mutation frequency in key oncogenes and tumor suppressors (*TP53*, *BRCA1*, *BRCA2*). Moreover, in contrast to the above-mentioned cell lines, the mRNA expression profiles of KURAMOCHI are similar to those of HGSOC [97]. A drawback of KURAMOCHI, however, is linked to the difficulties of its grafting into xenograft mouse models.

An emerging OC model linking the cellular and the animal models is the so-called “spheroids”. Spheroids are defined as 3D structure grown from stem cells and consisting of organ-specific cell types [99]. While spheroids can be originated from normal stem cells, they can be also originated from cancer cells [100]. OC spheroids, which recently became available [101,102], replicate the genomic and histological characteristics of the lesion from which they are derived. Being organized into a 3D structure, OC spheroids better resemble the in vivo structure of OC compared to 2D culture models. Thus, they are in principle more informative then 2D cultures when used to study the biology of OC and also drug effectiveness. Moreover, OC spheroids can be xenografted, thus allowing drug-sensitivity tests in vivo.

### 5.2. Animal Models

The only two non-human animals known to naturally develop OC are the egg-laying hen and the jaguar [103]. The egg-laying hen model has been underutilized due to the scarcity of chicken-specific experimental reagents and due to the logistical challenges of animal husbandry. Jaguar use has been largely impossible, mainly because these animals are endangered. Thus, the mouse represents the main animal model utilized so far to study anti-OC drugs. In mouse, three models are commonly used: the xenograft model, the syngenic model, and the genetically engineered mouse models (GEMM).

In xenograft mouse models, cells of different genetic backgrounds can be injected into immunocompromised animals via three different routes: subcutaneous (SC), intraperitoneally (IP), or intrabursally (IB, i.e., into the bursa that surrounds the mouse ovary) [104]. As in the SC approach, cells do not metastasize, this model is not suited to the study of ovarian metastasis. Moreover, the anatomical location/microenvironment of the injected cells is different from that in the ovary glands. However, the SC model is suited for investigation with imaging modalities; moreover, it is suitable for pilot studies to test the effectiveness of newly developed drugs. IP and IB better mimic the metastatic dissemination of OC. However, while IP cannot effectively reproduce the initial steps in metastasis (cells exit the bursa to spread throughout the peritoneal cavity), IB can. OC cell lines are most often employed to generate xenograft models. However, it is also possible to use cells freshly isolated from OC patients, thus resulting in models more adherent to reality. The problems with patients’ cells are often linked to the modest tumor engraftment percentages and the time taken to develop tumors [105]. Regardless of the cell type used, all xenograft mouse models suffer from the fact that the animals are immunocompromised; therefore, the role of the immune system is completely ignored.

A syngenic mouse model has been generated using murine ovarian surface epithelial cells from C57BL/6 mice, which have been subsequently transformed (ID8 cells) and eventually re-injected into C57BL/6 mice [106]. An alternative, more recent model, again derived from the ovarian surface epithelium, is the STOSE model generated in the FVB/N mice strain [107]. While it is possible to consider the effects of immune systems in these models, the cells used are different from human OC cells.

GEMMs of OC are useful for improving our understanding of the origin(s) of OCs. Indeed, it is possible to generate GEMMs with ovarian or oviductal epithelia carrying specific mutations. It is then possible to study how the specific mutation drives the development of OC. In this regards, GEMMs carrying the most common OC mutations, such as those of *TP53* and *BRCA1/2*, have been developed [104]. A problem liked to GEMMs is represented by the frequent infertility of GEMM animals. The other drawback stems from the fact that OCs tend to arise over an extended course of time, and thus, there are difficulties in controlling tumor onset and size.

### 5.3. Mathematical Models

An additional tool useful for the investigation and description of the effects and delivery of siRNA is based on the use of mathematical models. This approach, not yet very common in the field, has the possibility of predicting siRNA behavior once the main biological parameters have been established via an experimental approach. Thus, the system has the great advantage of significantly reducing the experimental load, as it can easily simulate/predict the effects of many variables of the system via mathematical simulation [108]. Interestingly, the mathematical approach is in line with Leonardo da Vinci’s conviction that “*niuna umana investigazione si può dimandare vera scientia s’essa non passa per le matematiche dimostrazioni*” (no human investigation can be defined true science if it cannot be mathematically demonstrated). [109]. In our opinion, the use of mathematical modeling in the siRNA field became possible after the publication of the seminal work of Bartlett and Davis [110]. Curiously, the mathematical modeling of siRNA delivery was not the only goal of that paper, but the mathematical model was also described in such a concise manner that it was very difficult to understand. The graphical translation of this model into two schemes [108] revealed its powerful theoretical basis. Indeed, this model, relaying on 12 ordinary differential equations, is organized into four modules that can be changed independently to modify model complexity as desired. The first module deals with siRNA “circulation/extracellular transport” (three equations), the second is about siRNA “cellular uptake and intracellular trafficking” (four equations), the third describes “the fate of free and bound activated RISC” (three equations) according to the Michaelis–Menten multiple turnover mechanism, and the fourth focuses on “cell growth and target protein production” (two equations). According to this modular organization, the model individuates two distinct phases: the external phase, involving the first module, and the cellular phase, involving the other three modules. Consequently, the description of in vivo experiments requires both phases, i.e., all four modules, while in vitro experiments imply the use of just the cellular phase, i.e., the last three modules. On the basis of this approach, Barlett and Davis [110] were able to fit and predict the silencing of a protein (luciferase) by means of siRNA. This clearly indicates the theoretical and practical strength of the model, which can help in optimizing (interpreting and planning) experimental activities.

## 6. siRNA Delivery to OC Cells

In the light of the above information/considerations (Section 1, Section 2, Section 3, Section 4 and Section 5), in this Section we describe and comment on the most recent works (from the last three years) related to the delivery of siRNAs based on the use of polymer-/lipid-based delivery systems. We concentrated on polymers and lipids due to their general biocompatibility, their ability to deliver siRNAs, and the possibility of their undergoing significant modifications of structure, a feature useful in generating optimized delivery materials. Polymeric approaches have been divided into systems: those which do not have an OC targeting strategy and those that do have one.

### 6.1. Polymeric Delivery Systems

#### 6.1.1. Non-Targeted Delivery Systems

**Figure 6:**
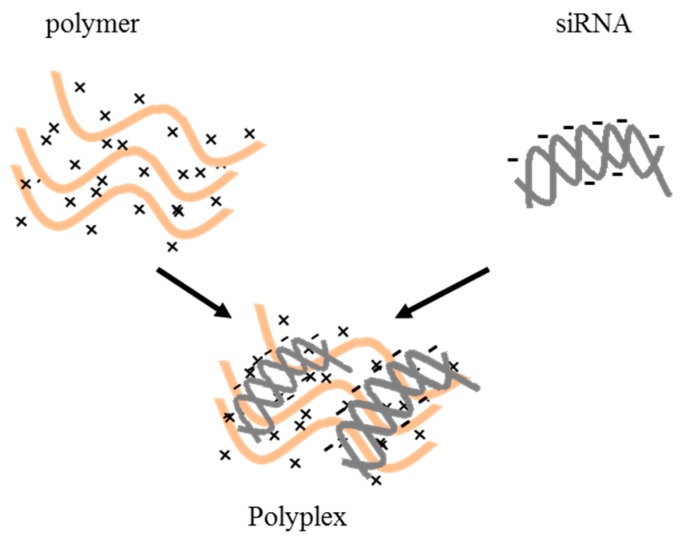
Polymeric delivery material developed in Reference [111]. Polymer: PGA conjugated with amine moieties.

Polyak et al. [111] developed a polymer comprising a poly(α)glutamate (PGA) backbone conjugated with different amine moieties (PGAamine) (Figure 6 and Table 5). Due to their positive electrostatic charge, amine groups allow interaction with the negatively charged siRNA. Moreover, the positive charge of the PGAamine polymer allows interaction with the negatively charged cell membrane. Finally, the multiple amino groups in acidic compartments (lysosomes, endosomes) may favor the proton sponge phenomenon, allowing the rupture of endocellular vesicle (lysosomes, endosomes) membranes, thus allowing siRNA release into the cytoplasm. siRNA release to the cytoplasm is also favored by the fact that the PGAamine polymer is cut by cathepsin B, an enzyme present in lysosomes/endosomes that it is upregulated in many human tumors. Upon cathepsin B cleavage of PGAamine, siRNAs are released from the delivery complex. Finally, PGA is water-soluble and has low immunogenicity and low toxicity; thus, it is an attractive candidate for siRNA release. As siRNA targets, the authors chose Rac1 (siRac1) and Plk1 (siPlk1), both involved in tumorigenesis via the control of cell migration and proliferation, respectively. A PGAamine:siRac1 polyplex demonstrated plasma stability and immune and hemo-compatibility in the ex vivo blood compartment. The functionality of PGAamine:siRac1 was proven in vitro in SKOV3 cells, showing the inhibition of cellular migration. In vivo, the authors used an orthotopic SKOV3 human OC model in athymic nude female mice. Following three sequential IP injections (8 mg/kg siRNA), a 2.75-fold increase in siRac1 tumor accumulation was observed compared to treatment with siRNA alone.

This was paralleled by a 44% decrease of Rac1 expression in tumor tissue compared to siRac1-alone-treated mice. The effect on tumor growth was studied using PGAamine:siPlk1 particles in nu/nu mice bearing orthotopic IP tumors of mCherry-labeled SKOV3 cells. Following nine daily IP injections of PGAamine:siPlk1 (8 mg/kg siRNA), tumor growth inhibition was 73% compared to control siRNA (siCtrl)-treated mice. Moreover, PGAamine:siPlk1 improved animal survival. Given the particle size (160 ± 20 nm) it is likely that most particles were cleared via the lymphatic lacunae (see Section 2.2). Thus, it is feasible to assume that the therapeutic effects were exerted by the fraction of particles which escaped lymphatic lacunae drainage and made direct contact with tumor cells in the peritoneum.

One of the major problems with HGSOC is the occurrence of drug resistance. Thus, the possibility of attenuating this eventuality has been explored using siRNA against MDR1 and BCL2, both involved in the induction of drug resistance (see Section 4.2). Risnayanti et al. [112] developed a PLGA-based delivery system loaded with siRNA against MDR1 (siMDR1) and BCL2 (siBCL2) (Table 5). To obtain an efficient siRNA encapsulation, siRNAs were first complexed with the cationic poly-l-lysine (PLL), which can bind to the negative charged siRNAs. PLL was used in place of the more commonly used PEI to minimize the known toxicity associated with PEI. siMDR1/siBCL2–PLL particles were then encapsulated with the negatively charged PLGA. The PLGA–siMDR1/siBCL2–PLL particles, with a dimeter of 197.8 ± 5.2 nm, did not elicit any nonspecific cytotoxicity in the drug-resistant SKOV3-TR and A2780-CP20 cells. In this regard, it is somewhat strange that particles containing the siBCL2 did not trigger any cell death, considering that *BCL2* is an anti-apoptotic gene. By in vitro test, the authors also showed that the particles did not stimulate the innate immune system. The efficiency of particle uptake was studied by the use of cyanine5.5 fluorophore (Cy5.5) labeled siRNAs, and was 100% in both SKOV3-TR and A2780-CP20 cell lines. This was paralleled by a significant reduction in the mRNA and protein levels of both MDR1 and BCL2. From the functional point of view, PLGA–siMDR1/siBCL2–PLL particles were able to increase the permanence and concentration of PTX in the drug resistant SKOV3-TR and A2780-CP20. This resulted in an improved cell death compared to PTX alone. Notably, the use of particle carrying only one of the two siRNAs + PTX was less effective than the particle loaded with the two siRNAs +PTX. This suggests the importance of downregulating both inducers of drug resistance. Comparable results were obtained using cisplatin in place of PTX. While no in vivo data were provided, the data support the concept that it is, in principle, possible to improve drug sensitivity by targeting specific genes.

Classical PLGA–PEI particles were used by Hazekawa et al. [113] to deliver a siRNA (siGPC3) against Glypican-3 (GPC3), a member of the heparan sulfate proteoglycans, which has important functions in cellular signaling pathways, including cell growth (Table 5). While GPC3 is over expressed in chemo-resistant OC cells, its overexpression seems to occur mostly in clear OC cell adenocarcinoma rather than in HSGOC [114]. In vitro in the mouse HM-1 cells, PLGA–PEI–siGPC3 particles efficiently reduced GPC3 protein levels and reduced cell growth compared to controls. In a murine HM-1 peritoneal dissemination model, the IP injection of PLGA–PEI–siGPC3 resulted in a significant reduction in the tumor nodules compared to controls. Moreover, the GPC3 levels in the cell lysates of peritoneal cells from animals treated with PLGA–PEI–siGPC3 were also reduced compared to control. While the role of GOC3 in HSGOC is not completely clear, a merit of this work was the use of the HM-1 cell line in the syngeneic B6C3F1 mice strain, which allowed consideration of the contribution of the immune system.

A complex polymer-based delivery system based on poly(vinyl benzyl trimethylammonium chloride) (PVTC) and its block copolymer with poly(oligo(ethyleneglycol) methacrylate) (POEGMA) was developed by Luo et al. [115] (Table 5). PVTC was used as it contains positively charged amino groups, which allow siRNA binding. The authors studied particles of PVTC or PVTC–POEGMA. The generated particles had a diameter range of 8–25 nm. In SKOV3-luc expressing the luciferase gene, PVTC–POEGMA and PVTC at the concentration of 15 μg/mL were significantly less toxic than PEI, used as a control polymer. Loading the particles with an anti-luciferase siRNA (luc-siRNA), the authors observed that at the concentration of 200 nM luc-siRNA and at a nitrogen/phosphate (N/P, polymer/siRNA) of 16, PVTC/luc-siRNA particles were the most effective in reducing luciferase expression (60% reduction) compared to control. Notably, PVTC/luc-siRNA particles were more effective than POEGMA/PVTC/luc-siRNA particles (35% inhibition). This difference is most probably due to the reduced cellular uptake of POEGMA/PVTC/luc-siRNA compared to PVTC/luc-siRNA, visualized by using a fluorescently labeled siRNA. No in vivo data were provided. However, the size of the particles (8–25 nm) suggests that in vivo these particles, when IP injected, could have been preferentially drained by the blood vessel (see Section 2.2). It remains to be determined whether this could be an appropriate route by which to reach metastatic tumor cells in the peritoneum.

Leung et al. [116] proposed an original strategy to combat OC. The authors found that cancer-associated fibroblasts upregulate the lipoma-preferred partner gene (LPP) in tumor endothelial cells. LPP expression levels in intra-tumor endothelial cells correlate with survival and chemo-resistance in OC patients. Moreover, LPP increases endothelial cell motility and tumor vessel leakiness. The author reasoned that the targeting of LPP by a siRNA (siLLP) could reduce tumor vessel amount and leakiness, thus increasing PTX permanence in the tumor site. In an orthotopic model of OC generated with luciferase-labeled OVCA432 cells, the authors delivered intravenous (IV) siLLP embedded into chitosan particles (CH–siLLP) together with PTX. The levels of LPP in tumor mass of animals treated with CH–siLLP were significantly lower than in controls. Moreover, the same animals had reduced tumor burdens and microvessel densities. This last observation suggests that LPP silencing reduced tumor angiogenesis. Finally, the authors demonstrated that tumor vessel leakiness was also reduced by CH–siLLP, and that this was paralleled by an increased PTX bioavailability. Notably, the combination of CH–siLLP with PTX gave a more pronounced reduction in tumor mass than the single treatment with either PTX or CH–siLLP, thus suggesting an additive effect of the combined treatment.

#### 6.1.2. Targeted Delivery Systems

**Figure 7:**
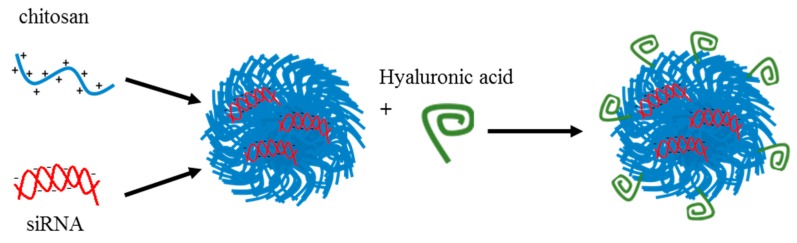
Polymeric delivery material developed in Reference [117].

The last example of the above section shows that it is reasonable to attack OCs via the targeting of tumor endothelial cells. An additional strategy to target tumor endothelial cells was described by Kim et al. [117] (Figure 7 and Table 6). The authors generated chitosan particles (CH–siRNA) coated with HA (HA–CH–siRNA) to target the CD44 receptor present on tumor endothelial cells. Particles were loaded with siRNA against PLXDC1 (siPLXDC1), which is involved in the promotion of cell migration and invasion of tumor endothelial cells (see also Section 4.2). PLXDC1 and CD44 are overexpressed in OC-associated endothelial cells [117]. In vitro, the authors showed that in two models of endothelial cells (HUVEC and MOEC), *PLXDC1* silencing by HA–CH–siPLXDC1 resulted in a significant inhibition of cell migration, invasion, and tube formation compared to particles carrying a control inactive siRNA. Using a Cy5-labeled siRNA, it was also possible to show that HA–CH–Cy5 siRNA efficiently entered the MOEC (CD44-positive) but not the A2780 OC model cells (CD44-negative). In an IP mouse model of OC generated with A2780 cells, HA–CH–Cy5 siRNA delivered IV (150 mg/kg) had improved tumor localization compared to CH–Cy5 siRNA, indicating the targeting role of HA. Using the HA–CH–siPLXDC1 particles (150 mg/kg, IV twice per week), the authors showed a significant decrease in the level of *PLXDC1* mRNA in the tumor mass compared to CH–control siRNA and CH–siPLXDC1. Unfortunately, no data about the effects on animal survival were reported. Despite this, as A2780 are CD44-negative and do not over express PLXDC1, the data presented suggest that the therapeutic effect was mostly due to an anti-angiogenic action. In a variation of the above in vivo test, the authors used an IP mouse model of OC generated with HeyA8 cells, which overexpress CD44 and PLXDC1. The results were quantitatively comparable to those obtained using the A2780. In this second test, it is reasonable to assume that the HA–CH–siPLXDC1 particles could have targeted both tumor endothelial cells and OC HeyA8 cells. Thus, an additive/synergistic effect could have been expected. However, this was neither visible from the data provided, nor did the authors discuss this aspect. Finally, it would have been interesting to test the developed vector in a drug-resistance model of OC, as endothelial cell targeting could be an alternative strategy to bypass drug resistance.

Byeon et al. [118] described the use of PLGA conjugated with HA (PLGA–HA) to target OC cells overexpressing CD44 (see Section 4.1) (Table 6). The authors used a siRNA directed against FAK (siFAK), which is overexpressed in OC, correlates with shorter overall patient survival, and promotes drug resistance (see Section 4.2). In addition to the siFAK, PLGA–HA also contained PTX, commonly used in OC therapy (see Section 1.3). The authors proved the effective uptake of fluorescently labeled PLGA–HA particles in the CD44 overexpressing HeyA8 and SKOV3 cells. In the HeyA8 and SKOV3 variants which are PTX resistant (HeyA8-MDR1 and SKOV3 TR), the authors showed that the presence of siFAK improved the effects of PTX. Effective tumor cell uptake in vivo was proven following a single IV injection of FITC-labeled HA–PLGA–NPs into HeyA8-bearing female BALB/c nude mice. Effectiveness studies were conducted in HeyA8-MDR and SKOV3-TR orthotopic models of OC, treating the animals twice weekly via IV injection of 200 mg/kg siFAK and 1.4 mg/kg PTX. In both OC animal models, the PLGA–HA–siFAK–PTX showed reduction of tumor weight, number of tumor nodules, and levels of FAK mRNA compared to controls. Notably, particles lacking HA invariably showed a reduced effect compared to those bearing HA, thus proving the targeting relevance of HA. Unfortunately, the authors did not compare the effects of PLGA–HA–siFAK–PTX with PLGA–HA–PTX; this test could have provided information about the role of siFAK alone in the therapeutic effect. This comparison was limited to the effects on animal survival, which indicated extended animal survival for PLGA–HA–siFAK–PTX compared to PLGA–HA–PTX. The authors also confirmed the effectiveness of their approach by loading the particles with a different siRNA against survivin (siSVV), a gene product known to induce chemo-resistance and to protect cells from apoptosis (see Section 4.2). The authors further confirmed their findings by testing the PLGA–HA–siFAK–PTX particles in a mouse model generated using OC cells derived from a patient that had developed PTX–carboplatin resistance. Altogether, these findings support the robustness of the approach undertaken.

A rather novel approach to particle targeting to OC was developed by Hong et al. [119]. The strategy was based on the equipment of PEG–PEI particles with a short peptide mimicking the sequence of the follicle-stimulating hormone (FSH) (Table 6). FSH is secreted by the pituitary gland and exerts its action on ovaries via the FSH receptor (FSHR). As FSHR is expressed at high levels in reproductive tissues and at very low levels in other tissues, FSH-equipped particles can almost be directed to the ovarian tissue. Notably, approximately 70% of ovarian cancers express FSHR [120]. The authors loaded the PEG–PEI–FSH particles with a siRNA directed against the growth-regulated oncogene α (gro-α), (sigro). Gro-α induces malignant transformation, tumor growth, and metastatic spread in OC cells, where it is overexpressed compared to healthy cells [121]. As the cellular model, Hey cells, which express both FSHR and gro-α, were employed. As controls, SKOV3 cells, which have low FSHR expression but elevated gro-α, were used. In vitro, the PEG–PEI–FSH–sigro reduced the target mRNA level down to 47.3% of that of the control. Notably, particles lacking the FSH peptide (PEG–PEI–sigro) were less effective (66.3% reduction). In the FSHR-negative SKOV3, PEG–PEI–FSH–sigro reduced target level down to only 79%, indicating the effectiveness of the targeting strategy. In Hey cells, the PEG–PEI–FSH–sigro particles more effectively reduced cell proliferation, migration, and invasion compared to control. In an in vivo xenograft subcutaneous mouse model of OC (Hey cell grafted), the IV administration of PEG–PEI–FSH–sigro particles resulted in a significant reduction in tumor mass compared to saline and empty-particle-treated animals. Unfortunately, no control siRNA was included in tests; thus, it is not possible to evaluate the specificity of the sigro chosen.

A commonly used targeting strategy in siRNA delivery system is based on the addition of FA, which can interact with FR, to the delivery material (see also Section 4.1). Jones et al. [122] prepared triblock copolymers consisting of hyperbranched polyethylenimine-graft-polycaprolactone-block-poly(-ethylene glycol) (hyPEI-*g*-PCL-*b*-PEG) with and without a FA ligand for FR targeting (Table 6). In vitro in SKOV3 cells, the authors observed that the polymer with or without FA, and loaded with indium-111 labeled siRNA, had comparable results in term of cellular uptake. This was mostly because uptake was evaluated just 4 h after treatment. Despite this, the cellular distribution in the presence or absence of FA influenced siRNA localization. Internalization via FR resulted in an even distribution of the siRNA within the cells, most likely due to the receptor-mediated internalization mechanisms. In the absence of FA, the distribution was dotted, probably reflecting the accumulation in endosomes, typical of an adsorptive endocytosis. In vivo, in an orthotropic model of OC, obtained using SKOV3 expressing the luciferase gene (SKOV3-luc), it was observed that when injected IV (35 μg of siRNA), the particles had short circulation half-lives. This was true for both the FR-targeted and -non-targeted particles, and was probably the consequence of the fast extravasation with predominant accumulation in the liver. FR-targeted particles displayed only a somewhat reduced liver localization compared to FR-non-targeted particles (38% and 53% of normalized injected dose per gram, respectively). Consequently, tumor accumulation was low and was characterized by an only slightly higher value for FR-targeted particles vs. -non-targeted (3.4 and 2.4%, respectively). When injected IP (35 μg of siRNA), both FR-targeted and -non-targeted particles accumulated predominantly in the kidney (7.36% and 7.78%, respectively) and in the tumor mass (5.63 and 5.28%, respectively). These data suggest that the effect of FR targeting was minimal, and tumor accumulation was most likely driven by passive targeting. From a functional point of view, the authors observed that the injection of an anti-luciferase siRNA delivered via the FR-targeted particles resulted in a significant reduction of luciferase expression 24 and 48 h post IP administration (35 μg siRNA-luc) compared to a control siRNA. From one point of view, this paper attenuated the enthusiasm for targeted delivery systems. On the other hand, the fact that just a small fraction of the siRNA reaching the tumor was sufficient to exert a clear anti-tumor effect is encouraging.

### 6.2. Lipid-Based Delivery Systems

Lee et al. [123] used particles comprising DC–Chol cationic lipids and DOPE neutral lipids for siRNA delivery (Table 7). To overcome the problems of short blood circulation time and potential aggregation of cationic lipids, the authors added PEG molecules to the lipid components (lipid–PEG). A siRNA against the kinesin spindle protein (siKSP) was used. KSP regulates microtubule organization and spindle assembly during eukaryotic cell division [124]; thus, its targeting can abrogate cell proliferation. Using a Cy5.5-labeled siRNA, it was possible to show that the lipid–PEG–Cy5.5–siRNA efficiently entered the cytoplasm of SKOV3 cells, but not the nucleus. Moreover, the lipid–PEG–Cy5.5–siKSP was able to efficiently reduce the mRNA and protein level of the target, as well as the proliferation of SKOV3. Following the IV injection of lipid–PEG–Cy5.5–siKSP in a subcutaneous xenograft model of OC, the authors observed an accumulation of the particles in the kidney and liver, with a detectable amount also found in the tumor mass. Notably, the particles did not elicit immunological activation, at least with regard to the levels of the cytokines TNF-α and IFN-α 1, 1 h post injection. Finally, lipid–PEG–siKSP reduced tumor mass growth compared to control. Unfortunately, no data about the effects on animal survival were reported. While the approach is, in principle, interesting, it was not determined whether KSP can be considered an appropriate target in OC treatment.

**Figure 8:**
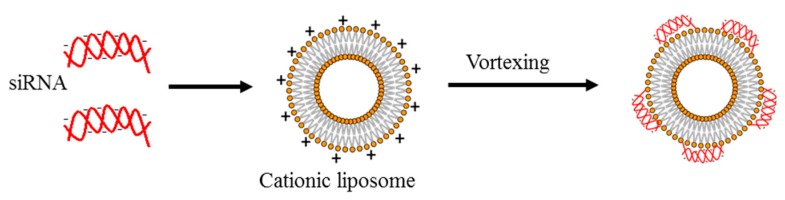
DOPC, DOPE, and DC delivery material developed in Reference [126].

An interesting molecule to target in OC is thymidylate synthase (TS). This is an important rate-limiting enzyme in both normal and tumor DNA biosynthesis. Moreover, TS is highly expressed in both original and metastatic (peritoneal) OC lesions [125]. Its expression level seems to also be a key determinant for the efficacy of TS-targeting drugs. Iizuka et al. [126] developed delivery particles comprising dioleoylphosphatidylcholine (DOPC), DOPE, and DC, which were loaded with an anti-TS siRNA, giving origin to the preparation DFP-10825 (Figure 8 and Table 7). The effects of DFP-10825 alone or in combination with PTX were tested in a xenograft orthotopic model of OC generated by SKOV3-luc implantation in the peritoneum. Up to 24 h from IP administration of DFP-10825 (1 mg/kg of TS siRNA), the anti-TS siRNA could be detected in the ascitic fluids, with a pick of 4.26 ± 1.00 nM at 2 h. Notably, at the same time point, TS siRNA in the blood only reached the concentration of 0.005 ± 0.002 nM. Given the average size of DFP-10825 (395 ± 32 nm), this result is not surprising. Indeed, particles with diameters greater than 10 nm are scarcely drained by the blood vessels (see Section 2.2). Verified the favorable retention of DFP-10825 in the ascitic fluids, the authors tested the efficacy and found that the IP injection of DFP-10825 (0.5–2 mg/kg every third day) resulted in a time- and dose-dependent reduction of tumor mass. Unfortunately, no control siRNA was used, and thus the real specificity of the siRNA remains undetermined. No variation in animal weight was observed over the 28 days of the experiment, thus suggesting mild toxicity of DFP-10825. In an additional experiment, PTX at a dose of 15 mg/kg was IP administered alone or in combination with DFP-10825. Due to the potent antitumor activity of PTX alone, addition of DFP-10825 only resulted in a modest augmentation of antitumor activity. The antitumor effects resulted in an increased animal survival for both DFP-10825- or PTX-treated animals. Notably, the combination of DFP-10825 and PTX produced an additive effect on animal survival. Finally, the authors proved that DFP-10825, but not PTX, reduced TS mRNA levels in tumor cells collected from ascites.

While lipid particles most often contain lipids only, non-lipid molecules are sometimes added to improve the delivery properties. This was the case in the work presented by Mendes et al. [127] (Table 7). The authors developed lipid particles containing phosphatidylcholine (PC), Chol, and 1,2-dioleoyl-*sn*-glycero-3-phosphoethanolamine-*N*-(glutaryl) (NGPE) covered by low-molecular-weight branched PEI (PEIPOS). Three kinds of particles were prepared: PEIPOS alone, and PEIPOS with 0.1% and 0.5% by moles of PEI on the surface. PEI was added to improve siRNA delivery (see Section 3.1). Compared to PEIPOS and 0.1%PEIPOS, 0.5%PEIPOS particles had the highest uptake in A2780-ADR and SKOV3-TR cells, resistant to the drugs adriamycin and PTX, respectively. Most likely, the higher PEI amount in 0.5%PEIPOS was better able to exteriorize PEI delivery properties. In 3D tumor spheroids generated from cervical adenocarcinoma HeLa cells (not related to OC), 0.5%PEIPOS association to the tumor cells was higher (88%) than PEIPOS and 0.1%PEIPOS. Moreover, the authors showed that 0.5%PEIPOS could penetrate deeper into a spheroid structure by inter- and intra-cellular routes. Although HeLa spheroids are not directly pertinent to OC study, these data are encouraging for further tests in OC spheroids. In cultured A2780-ADR cells PEIPOS, 0.1%PEIPOS, and 0.5%PEIPOS loaded with PTX improved cytotoxicity. However, no significant differences were noted between PEIPOS and 0.5%PEIPOS, leaving open the possibility that PEI might not have been essential for siRNA delivery in the specific experimental model. In the same cellular model, 100 nM siMDR1 (N/P of 13) coupled with 0.5%PEIPOS reduced the target protein level by about 20%. An in vivo model, a xenograft subcutaneous mice model generated using A2780-ADR, was employed. Animals were treated continuously with 0.5%PEIPOS/PTX/siMDR1 on alternate days until 12 injections has been administered, reaching the total doses of 66 mg/kg and 9.6 mg/kg of PTX and siMDR1, respectively. It is unclear why the authors used A2780-ADR in combination with PTX and not adriamycin, for which these cells show resistance. Only the 0.5%PEIPOS/PTX/siMDR1 treatment resulted in a reduction of tumor growth compared to free PTX and 0.5%PEIPOS/PTX. The authors suggested that the reason for the lack of effectiveness of the 0.5%PEIPOS/PTX may have been in the low dose used. Unfortunately, 0.5%PEIPOS/PTX/sicontrol was not tested; thus, the specificity of the siRNA remains undetermined. Moreover, 0.5%PEIPOS/siMDR1 was not tested, so it is not possible to evaluate the effects of the siRNA per se. Finally, the 0.5%PEIPOS/PTX/siMDR1 treatment had no significant side effects as evaluated by animal weight and by the levels of transaminases.

Recently, pressurized IP aerosol chemotherapy (PIPAC) has been studied as a novel drug delivery method in the treatment of OC peritoneal metastasis. Therapeutic drugs are nebulized throughout the peritoneal cavity via the use of high pressure (≈20 bars). This is thought to guarantee a homogenous distribution of the aerosolized particles containing the drug in the peritoneal cavity [128]. Minnaert et al. [129] studied the effects of the nebulization on a lipid (commercial) complex carrying siRNA against the luciferase gene, siLuc (Table 7). The authors observed that, from a physical point of view, the nebulization process only resulted in a small increase in particle size (about 25%, from 141 ± 1 to 193 ± 8). However, using particles loaded with up to 2 pmol of siLuc, the ability to reduce the fluorescence of SKOV3-luc in vitro was decreased. This phenomenon was not observed using higher siLuc amounts and, in general, the authors observed a dose-dependent reduction in the luminescence of SKOV3-luc. To mimic the real pathological environment, particles were incubated with ascitic liquid. The authors observed that the incubation reduced siLuc effectiveness by about 20%. The authors suggested that this could have been due to the binding of negatively charged proteins of ascites to the particles. Alternatively, the ascites’ protein content might have influenced the intracellular pathway of siRNA. While no data in an animal model were provided, the work suggests that for lipid–siRNA complexes, delivery via nebulization in the peritoneal cavity might be feasible.

## 7. Final Considerations

The use of naked siRNA in vivo results in negligible effects due to their instability in biological fluids and sub-optimal pharmacokinetics [130,131,132]. The use of polymer-/lipid-based materials has the potential to bypass the above problems, allowing the development of siRNA delivery systems suitable for therapeutic applications. Despite this, almost all the works published so far (1930 publications found in PubMed using the key words: “ovarian cancer siRNA”) tested the delivery systems in orthotopic mouse models of OC at best. We could find only one clinical trial (NCT01437007) mentioning the use of siRNA in OC [133]. In this case, the aim was to evaluate the effect of a lipid particle formulation containing siRNA against PLK1 (TKM-080301) in patients with liver metastases from different tumors, including OC. Following hepatic arterial administration (4 mg/m^2^ every 2 weeks for up to 12 doses) the study aimed to establish the maximum tolerable dose and dose-limiting toxicities. Although the clinical trial was posted in 2011, no conclusive results have been reported so far.

Based on the above consideration, it is evident that improvements in the delivery techniques available are necessary. In this regard, different factors can be considered work towards improved delivery effectiveness.

The first deals with the use of appropriate models for OC. Most of the work published used cellular models, which do not share the phenotypic/molecular features of HGSOC. Thus, the results obtained can only barely be extrapolated to HGSOC. It is therefore advisable to use more appropriate cell models in the future [97], possibly in the form of spheroids that better resemble in vivo situations. The problem of suboptimal animal models is harder to circumvent. However, the recommendation is to use orthotopic mouse models, which better resemble the peritoneal metastasis observed in humans even if most cannot take into account the effects of the immune system. The usefulness of subcutaneous mouse models, where the effects on tumor mass are easily measurable, might be limited to pilot studies for the preliminary testing of completely novel drugs and/or delivery systems. To further improve the usefulness of cellular/animal models, we believe that the use of mathematical models should be more thoroughly considered. Indeed, through mathematical models, it is possible to reduce experimental testing, with the consequent reduction in experimental burden and costs.

A second factor involves the possibility of targeting siRNA to OC cells. This approach may, in principle, allow normal cells to be left untouched, thus minimizing side effects. However, targeting is not so easy to achieve. One reason for this is that the targeted antigen is often present (albeit at lower levels) also on non-tumor cells, and it remains unclear whether this level of difference is sufficient for effective discrimination. To improve the specificity, one possibility is to combine a targeted delivery system with an siRNA directed against the mRNA of genes expressed only or predominantly in OC cells. In this way, even if the siRNAs reach healthy cells, they cannot exert major detrimental effects, as the target gene is expressed at low or negligible levels. Despite the above considerations, OC targeting may be more difficult to achieve than we think. For example, the work of Jones et al. [122], discussed above, showed that targeted/non-targeted particles behave very similarly with regard to the body distribution when delivered via either IV or IP administration. Additionally, tumor accumulation is modest for both methods, most likely due to passive targeting. It is comforting, however, that even a modest tumor accumulation seems to be sufficient to exert a functional effect. Moreover, evidence of the possibility of a benefit from target delivery systems has been reported in References [117,118], commented on in this review.

A third factor to be considered is the optimal size of the vector delivery particles. In the case of IV administration, it is known that particles of 1–3 μm diameter tend to localize closer to the endothelial layer (margination effect) of the vessel, whereas smaller particles localize to the middle of the vessel [134]. Thus, 1–3 μm particles, being closer to the vessel wall, have a better chance to extravasate. However, particles in the nm range are more suited to crossing cell membranes compared to μm particles. Thus, a possible compromise might involve the preparation of μm particles that, upon extravasation, undergo fragmentation to generate nm-sized particles (chimeric systems). Notably, in all the works described here where IV administration was chosen, the particle size was 150–200 nm (Table 5, Table 6 and Table 7), i.e., not optimized for the margination effect and thus extravasation. In the case of IP administration, the retention of delivery systems in the peritoneal cavity is a function of the particle size. Delivery systems larger than the diameter of the aperture of the lymphatic lacunae (3.2–3.6 μm in humans) will persist longer in the peritoneal cavity. Particles with smaller size will be predominantly cleared via the lacunar route, and the very small particles (nm range) will be mostly drained by blood vessels. In the works described where the IP route was chosen (Table 5, Table 6 and Table 7), particle size was 100–395 nm, and thus not optimal for long peritoneum persistence. Additionally, a chimeric system composed of μm particles which can dissociate into nm particles route may allow proper peritoneum persistence and subsequently the crossing of tumor cell membrane with IP administration.

A final factor, which should be considered to improve the effectiveness of delivered siRNAs, deals with biological aspects, i.e., the targeting of OC stem cells (OCSCs). Almost all the published studies to date have focused their attention on general OC cells. However, targeting OCSCs would represent an attractive strategy, as these cells have the ability to perpetuate the tumor, thus favoring its persistence and spread. Moreover, OCSCs seem to have the ability to escape chemotherapy [69]. To develop such a strategy, the precise recognition of OC stem cells would be necessary, a problem which does not seem too complex, as different antigens for OC stem cells are known (see Section 4.1).

In conclusion, although the above issues may dampen enthusiasm for future research in the OC area with regard to siRNAs, the development of effective delivery systems should be further pursued. In particular, we believe that by taking into account the physiology and molecular biology of peritoneum/vessel/OC cells, it is possible to properly optimize siRNA delivery systems for OC.

## Figures and Tables

**Table 1:** Specific surface antigens on OC cells.

Extended Name	Abbreviation	References
Epidermal growth factor receptor	EGFR	[61,62,63,64]
Erythropoietin-producing hepatocellular receptor A2	EphA2	[65,66]
Folic acid receptor	FR	[67,68]
CD44 surface transmembrane glycoprotein	CD44	[69,70,71]
CD133 glycosylated transmembrane protein	CD133	[72]
CD117 (also known as c-kit) receptor tyrosine kinase	CD117	[73,74,75]
CD24	CD24	[76]

**Table 2:** Molecular targets implicated in OC cell growth/migration.

Extended Name	Abbreviation	References
*Epidermal growth factor receptor*	*EGFR*	[64]
*Metastasis associated in colon cancer 1*	*MACC1*	[77]
*Metastasis-associated gene 1*	*MTA1*	[78]
*Wilms tumor gene*	*WT1*	[79]
*Rac1 Rho family small GTPases*	*Rac1*	[80,81]
*Polo-like kinase 1*	*Plk1*	[82,83]
*Notch1* *Claudin3* *Nin one binding protein* *Cyclooxigenase-2*	*Notch1* *CLDN3* *NOB1p* *COX-2*	[61]
*E2 promoter binding factor 1*	*E2F1*	[84,85,86,87]
*Peptidylprolyl cis–trans isomerase, NIMA-interacting 1*	*PIN1*	[88,89]

**Table 3:** Molecular targets implicated in angiogenesis.

Extended Name	Abbreviation	References
Vascular endothelial growth factors	VEGFs	[61]
VEGFs tyrosine kinase receptors	VEGFR-1/Ftl-1, VEGFR-2, VEGFR-3/Ftl-4	[61]
Plexin domain containing 1	PLXDC1	[90]

**Table 4:** Molecular targets implicated in drug resistance.

Extended Name	Abbreviation	References
*Multidrug resistance gene 1*	*MDR1*	[91]
*Survivin*	*SVV*	[92,93]
*Focal adhesion kinase*	*FAK*	[94]
*B-cell lymphoma 2*	*BCL2*	[95]

**Table 5:** Polymer based delivery systems without an OC-targeting moiety.

First Author	Target	Delivery Material	Cell Model	siRNA Delivery Route	Animal Model	References
Polyak	Rac1; Plk1	Poly(α)glutamate; no specific targeting; 160 ± 20 nm (Particle size)	SKOV3	Intra-peritoneal (nine every other day intraperitoneal injections 8 mg/kg siRNA)	Orthotopic SKOV3 cells in athymic nude female mice (intra-peritoneal tumor cell injection)	[111]
Risnayanti	MDR1; BCL2	PLGA–PLL; 197.8 ± 5.2 nm (Particle size)	SKOV3-TR and A2780-CP20	-	-	[112]
Hazekawa	Gpc3	PLGA–PEI; 108.5 ± 2.5 nm (Particle size)	HM-1	Intra-peritoneal (100 pmol of siRNA on Day 1)	Syngeneic orthotopic intra-peritoneal HM-1 injection in syngeneic B6C3F1 mouse strain	[113]
Lou	Luciferase	POEGMA/PVTC; PVTC; 8–25 nm (particle sizes)	SKOV-3-luciferase	-	-	[115]
Leung	LPP	CHITOSAN	Luciferase-labeled OVCA432	Intravenous (twice-weekly tail-vein 5 μg siRNA in combination with weekly i.p. injections of Paclitaxel, 3.5 mg/kg for 6 weeks)	Orthotopic luciferase-labeled OVCA432 in female nude mice (intra-peritoneal tumor cell injection)	[116]

**Table 6:** Polymer based delivery systems with an OC-targeting moiety.

First Author	Target	Delivery Material	Cell Model	siRNA Delivery Route	Animal Model	References
Kim	PLXDC1	HA–CHITOSAN (targeting to CD44) 200 ± 10 nm (particle size)	HUVEC; MOEC; A2780; HeyA8	Intravenous (150 mg/kg twice per week)	Orthotopic A2780, HeyA8- Female BALB/c nude mice (intra-peritoneal tumor cell injection)	[117]
Byeon	FAK, surviving	HA–PLGA (targeting to CD44) 200–220 nm (particle size)	HeyA8; SKOV3; HeyA8-MDR; SKOV3-TR	Intravenous (200 mg/kg siFAK and 1.4 mg/kg PTX)	Orthotopic HeyA8-MDR, SKOV3-TR, patient-derived cells in female BALB/c nude mice (intra-peritoneal tumor cell injection)	[118]
Hong	Gro-α	PEG–PEI–FSH (targeting FSHR) 142.0 ± 3.8 nm	Hey	Intravenous (5 mg/kg)	Subcutaneous xenograft Hey BALB/c nude mice	[119]
Jones	Luciferase	hyPEI-g-PCL-b-PEG with and without FA 150 nm (particle size)	SKOV3	Intravenous (35 μg siRNA) Intra-peritoneal (35 μg siRNA)	Orthotopic SKOV3female nude mice (intra-peritoneal tumor cell injection)	[122]

**Table 7:** Lipid-based delivery systems.

First Author	Target	Delivery Material	Cell Model	siRNA Delivery Route	Animal Model	References
Lee	KSP	DC–Chol–DOPE–PEG 90–110 nm (Particle size)	SKOV3	Intravenous (1 mg/kg every other day for a total of eight injections)	Subcutaneous xenograft mice model Balb/c nude mice	[123]
Iizuka	TS	DOPC–DOPE–DC 395 ± 32 nm (Particle size)	-	Intra-peritoneal (0.5–2 mg/kg every third day)	Orthotopic SKOV3-luc cell in male SOD/SCID mice (intra-peritoneal tumor cell injection)	[126]
Mendes	MDR1	PC–Chol–NGPE–PEI 161 ± 9.4 nm (Particle size)	A2780-ADR and SKOV3-TR	Intravenous (total doses in multiple administration: 66 mg/kg and 9.6 mg/kg of PTX and siMDR1, respectively)	Subcutaneous xenograft mice (athymic nude mice) model using A2780-ADR	[127]
Minnaert	luciferase	Commercial lipid 193 ± 8 nm (Particle size) Following nebulization	SKOV3-luc	-	-	[129]

## References

[1] Farra R., Dapas B., Pozzato G., Scaggiante B., Agostini F., Zennaro C., Grassi M., Rosso N., Giansante C., Fiotti N. (2011). Effects of E2F1–cyclin E1–E2 circuit down regulation in hepatocellular carcinoma cells. Dig. Liver Dis..

[2] Grassi M., Cavallaro G., Scirè S., Scaggiante B., Daps B., Farra R., Baiz D., Giansante C., Guarnieri G., Perin D. (2010). Current Strategies to Improve the Efficacy and the Delivery of Nucleic Acid Based Drugs. Curr. Signal Transduct. Ther..

[3] Grassi G., Dawson P., Guarnieri G., Kandolf R., Grassi M. (2004). Therapeutic potential of hammerhead ribozymes in the treatment of hyper-proliferative diseases. Curr. Pharm. Biotechnol..

[4] Grassi G., Marini J.C. (1996). Ribozymes: Structure, function, and potential therapy for dominant genetic disorders. Ann. Med..

[5] Grassi G., Schneider A., Engel S., Racchi G., Kandolf R., Kuhn A. (2005). Hammerhead ribozymes targeted against cyclin E and E2F1 cooperate to down-regulate coronary smooth muscle cell proliferation. J. Gene Med..

[6] Goldberg M.S. (2013). siRNA delivery for the treatment of ovarian cancer. Methods.

[7] Van den Brand D., Mertens V., Massuger L.F.A.G., Brock R. (2018). siRNA in ovarian cancer—Delivery strategies and targets for therapy. J. Control. Release.

[8] Aghamiri S., Mehrjardi K.F., Shabani S., Keshavarz-Fathi M., Kargar S., Rezaei N. (2019). Nanoparticle-siRNA: A potential strategy for ovarian cancer therapy?. Nanomedicine.

[9] Halbur C., Choudhury N., Chen M., Kim J.H., Chung E.J. (2019). siRNA-Conjugated Nanoparticles to Treat Ovarian Cancer. SLAS Technol..

[10] Webb P.M., Jordan S.J. (2017). Epidemiology of epithelial ovarian cancer. Best Pract. Res. Clin. Obstet. Gynaecol..

[11] Reid B.M., Permuth J.B., Sellers T.A. (2017). Epidemiology of ovarian cancer: A review. Cancer Biol. Med..

[12] Singh A., Gupta S., Sachan M. (2019). Epigenetic Biomarkers in the Management of Ovarian Cancer: Current Prospectives. Front. Cell Dev. Biol..

[13] Siegel R.L., Miller K.D., Jemal A. (2016). Cancer statistics, 2016. CA Cancer J. Clin..

[14] Chang S.J., Hodeib M., Chang J., Bristow R.E. (2013). Survival impact of complete cytoreduction to no gross residual disease for advanced-stage ovarian cancer: A meta-analysis. Gynecol. Oncol..

[15] Shih I., Kurman R.J. (2004). Ovarian tumorigenesis: A proposed model based on morphological and molecular genetic analysis. Am. J. Pathol..

[16] Verhaak R.G., Tamayo P., Yang J.Y., Hubbard D., Zhang H., Creighton C.J., Fereday S., Lawrence M., Carter S.L., Mermel C.H. (2013). Prognostically relevant gene signatures of high-grade serous ovarian carcinoma. J. Clin. Investig..

[17] Konecny G.E., Wang C., Hamidi H., Winterhoff B., Kalli K.R., Dering J., Ginther C., Chen H.W., Dowdy S., Cliby W. (2014). Prognostic and therapeutic relevance of molecular subtypes in high-grade serous ovarian cancer. J. Natl. Cancer Inst..

[18] Wang C., Armasu S.M., Kalli K.R., Maurer M.J., Heinzen E.P., Keeney G.L., Cliby W.A., Oberg A.L., Kaufmann S.H., Goode E.L. (2017). Pooled Clustering of High-Grade Serous Ovarian Cancer Gene Expression Leads to Novel Consensus Subtypes Associated with Survival and Surgical Outcomes. Clin. Cancer Res..

[19] Lisio M.A., Fu L., Goyeneche A., Gao Z.H., Telleria C. (2019). High-Grade Serous Ovarian Cancer: Basic Sciences, Clinical and Therapeutic Standpoints. Int. J. Mol. Sci..

[20] Narod S. (2016). Can advanced-stage ovarian cancer be cured?. Nat. Rev. Clin. Oncol..

[21] Nieman K.M., Kenny H.A., Penicka C.V., Ladanyi A., Buell-Gutbrod R., Zillhardt M.R., Romero I.L., Carey M.S., Mills G.B., Hotamisligil G.S. (2011). Adipocytes promote ovarian cancer metastasis and provide energy for rapid tumor growth. Nat. Med..

[22] Matulonis U.A., Sood A.K., Fallowfield L., Howitt B.E., Sehouli J., Karlan B.Y. (2016). Ovarian cancer. Nat. Rev. Dis. Primers.

[23] Gonzalez-Martin A., Sanchez-Lorenzo L., Bratos R., Marquez R., Chiva L. (2014). First-line and maintenance therapy for ovarian cancer: Current status and future directions. Drugs.

[24] Markman M. (2003). Optimizing primary chemotherapy in ovarian cancer. Hematol. Oncol. Clin. N. Am..

[25] Marchetti C.,  De Felice F., Perniola G., Palaia I., Musella A., Di Donato V., Cascialli G., Muzii L., Tombolini V., Cascialli G. (2019). Role of intraperitoneal chemotherapy in ovarian cancer in the platinum-taxane-based era: A meta-analysis. Crit. Rev. Oncol. Hematol..

[26] Bast R.C., Hennessy B., Mills G.B. (2009). The biology of ovarian cancer: New opportunities for translation. Nat. Rev. Cancer.

[27] Papa A., Caruso D., Strudel M., Tomao S., Tomao F. (2016). Update on Poly-ADP-ribose polymerase inhibition for ovarian cancer treatment. J. Transl. Med..

[28] Napoli C., Lemieux C., Jorgensen R. (1990). Introduction of a Chimeric Chalcone Synthase Gene into Petunia Results in Reversible Co-Suppression of Homologous Genes in trans. Plant Cell.

[29] Fire A., Xu S., Montgomery M.K., Kostas S.A., Driver S.E., Mello C.C. (1998). Potent and specific genetic interference by double-stranded RNA in Caenorhabditis elegans. Nature.

[30] Elbashir S.M., Harborth J., Lendeckel W., Yalcin A., Weber K., Tuschl T. (2001). Duplexes of 21-nucleotide RNAs mediate RNA interference in cultured mammalian cells. Nature.

[31] Lang C., Sauter M., Szalay G., Racchi G., Grassi G., Rainaldi G., Mercatanti A., Lang F., Kandolf R., Klingel K. (2008). Connective tissue growth factor: A crucial cytokine-mediating cardiac fibrosis in ongoing enterovirus myocarditis. J. Mol. Med..

[32] Grassi G., Pozzato G., Moretti M., Giacca M. (1995). Quantitative analysis of hepatitis C virus RNA in liver biopsies by competitive reverse transcription and polymerase chain reaction. J. Hepatol..

[33] Scaggiante B., Dapas B., Farra R., Grassi M., Pozzato G., Giansante C., Fiotti N., Grassi G. (2011). Improving siRNA bio-distribution and minimizing side effects. Curr. Drug Metab..

[34] Farra R., Grassi M., Grassi G., Dapas B. (2015). Therapeutic potential of small interfering RNAs/micro interfering RNA in hepatocellular carcinoma. World J. Gastroenterol..

[35] Grassi G., Scaggiante B., Dapas B., Farra R., Tonon F., Lamberti G., Barba A., Fiorentino S., Fiotti N., Zanconati F. (2013). Therapeutic potential of nucleic acid-based drugs in coronary hyper- proliferative vascular diseases. Curr. Med. Chem..

[36] Agostini F., Dapas B., Farra R., Grassi M., Racchi G., Klingel K., Kandolf R., Heidenreich O., Mercatahnti A., Rainaldi G. (2006). Potential applications of small interfering RNAs in the cardiovascular field. Drug Future.

[37] Huang Y., Hong J., Zheng S., Ding Y., Guo S., Zhang H., Zhang X., Du Q., Liang Z. (2011). Elimination pathways of systemically delivered siRNA. Mol. Ther..

[38] Jackson A.L., Linsley P.S. (2010). Recognizing and avoiding siRNA off-target effects for target identification and therapeutic application. Nat. Rev. Drug Discov..

[39] Sarfarazi A., Lee G., Mirjalili S.A., Phillips A.R.J., Windsor J.A., Trevaskis N.L. (2019). Therapeutic delivery to the peritoneal lymphatics: Current understanding, potential treatment benefits and future prospects. Int. J. Pharm..

[40] Trevaskis N.L., Kaminskas L.M., Porter C.J. (2015). From sewer to saviour—Targeting the lymphatic system to promote drug exposure and activity. Nat. Rev. Drug Discov..

[41] Mirahmadi N., Babaei M.H., Vali A.M., Dadashzadeh S. (2010). Effect of liposome size on peritoneal retention and organ distribution after intraperitoneal injection in mice. Int. J. Pharm..

[42] Lengyel E. (2010). Ovarian cancer development and metastasis. Am. J. Pathol..

[43] Luft C., Ketteler R. (2015). Electroporation Knows No Boundaries: The Use of Electrostimulation for siRNA Delivery in Cells and Tissues. J. Biomol. Screen..

[44] Leonetti J.P., Degols G., Lebleu B. (1990). Biological activity of oligonucleotide-poly(l-lysine) conjugates: Mechanism of cell uptake. Bioconjug. Chem..

[45] Sardo C., Farra R., Licciardi M., Dapas B., Scialabba C., Giammona G., Grassi M., Grassi G., Cavallaro G. (2015). Development of a simple, biocompatible and cost-effective Inulin-Diethylenetriamine based siRNA delivery system. Eur. J. Pharm. Sci..

[46] Cavallaro G., Licciardi M., Amato G., Sardo C., Giammona G., Farra R., Dapas B., Grassi M., Grassi G. (2014). Synthesis and characterization of polyaspartamide copolymers obtained by ATRP for nucleic acid delivery. Int. J. Pharm..

[47] Xu C., Wang J. (2015). Delivery systems for siRNA drug development in cancer therapy. Asian J. Pharm. Sci..

[48] Hobel S., Aigner A. (2013). Polyethylenimines for siRNA and miRNA delivery in vivo. Wiley Interdiscip. Rev. Nanomed. Nanobiotechnol..

[49] Liu L., Zheng M., Librizzi D., Renette T., Merkel O.M., Kissel T. (2016). Efficient and Tumor Targeted siRNA Delivery by Polyethylenimine-graft-polycaprolactone-block-poly(ethylene glycol)-folate (PEI-PCL-PEG-Fol). Mol. Pharm..

[50] Roberts M.J., Bentley M.D., Harris J.M. (2002). Chemistry for peptide and protein PEGylation. Adv. Drug Deliv. Rev..

[51] Bao Y., Jin Y., Chivukula P., Zhang J., Liu Y., Liu J., Clamme J.P., Mahato R.I., Ng D., Ying W. (2013). Effect of PEGylation on biodistribution and gene silencing of siRNA/lipid nanoparticle complexes. Pharm. Res..

[52] Muralidharan P., Mallory E., Malapit M., Hayes D., Mansour H.M. (2014). Inhalable PEGylated Phospholipid Nanocarriers and PEGylated Therapeutics for Respiratory Delivery as Aerosolized Colloidal Dispersions and Dry Powder Inhalers. Pharmaceutics.

[53] Azimi B., Nourpanak P., Rabiee M., Arab S. (2014). Poly (ε-caprolactone) Fiber: An Overview. J. Eng. Fibers Fabr..

[54] Xu Z., Wang D., Cheng Y., Yang M., Wu L.P. (2018). Polyester based nanovehicles for siRNA delivery. Mater. Sci. Eng. C Mater. Biol. Appl..

[55] Posocco B., Dreussi E., de Santa J., Toffoli G., Abrami M., Musiani F., Grassi M., Farra R., Tonon F., Grassi G. (2015). Polysaccharides for the Delivery of Antitumor Drugs. Materials.

[56] Ahmed T., Aljaeid B. (2016). Preparation characterization and potential application of chitosan, chitosan derivates, and chitosan metal nanoparticles in pharmaceutical drug delivery. Drug Des. Dev. Ther..

[57] Khan W., Hosseinkhani H., Ickowicz D., Hong P.D., Yu D.S., Domb A.J. (2012). Polysaccharide gene transfection agents. Acta Biomater..

[58] Oh E.J., Park K., Kim K.S., Kim J., Yang J.A., Kong J.H., Lee M.Y., Hoffman A.S., Hahn S.K. (2010). Target specific and long-acting delivery of protein, peptide, and nucleotide therapeutics using hyaluronic acid derivatives. J. Control. Release.

[59] Barba A.A., Lamberti G., Sardo C., Dapas B., Abrami M., Grassi M., Farra R., Tonon F., Forte G., Musiani F. (2015). Novel Lipid and Polymeric Materials as Delivery Systems for Nucleic Acid Based Drugs. Curr. Drug Metab..

[60] Bochicchio S., Dalmoro A., Barba A.A., Grassi G., Lamberti G. (2014). Liposomes as siRNA delivery vectors. Curr. Drug Metab..

[61] Ayen A., Jimenez M.Y., Marchal J.A., Boulaiz H. (2018). Recent Progress in Gene Therapy for Ovarian Cancer. Int. J. Mol. Sci..

[62] Hsu P., Jablons D., Yang C., You L. (2019). Epidermal Growth Factor Receptor (EGFR) Pathway, Yes-Associated Protein (YAP) and the Regulation of Programmed Death-Ligand 1 (PD-L1) in Non-Small Cell Lung Cancer (NSCLC). Int. J. Mol. Sci..

[63] Shepard H.M., Brdlik C.M., Schreiber H. (2008). Signal integration: A framework for understanding the efficacy of therapeutics targeting the human EGFR family. J. Clin. Investig..

[64] Dickerson E.B., Blackburn W.H., Smith M.H., Kapa L.B., Lyon L.A., McDonald J.F. (2010). Chemosensitization of cancer cells by siRNA using targeted nanogel delivery. BMC Cancer.

[65] Zhou Y., Sakurai H. (2017). Emerging and Diverse Functions of the EphA2 Noncanonical Pathway in Cancer Progression. Biol. Pharm. Bull..

[66] Takahashi Y., Hamasaki M., Aoki M., Koga K., Koshikawa N., Miyamoto S., Nabeshima K. (2018). Activated EphA2 Processing by MT1-MMP Is Involved in Malignant Transformation of Ovarian Tumours In Vivo. Anticancer Res..

[67] Cortez A.J., Tudrej P., Kujawa K.A., Lisowska K.M. (2018). Advances in ovarian cancer therapy. Cancer Chemother. Pharmacol..

[68] Cheung A., Bax H.J., Josephs D.H., Ilieva K.M., Pellizzari G., Opzoomer J., Bloomfield J., Fittall M., Grigoriadis A., Figini M. (2016). Targeting folate receptor alpha for cancer treatment. Oncotarget.

[69] Zong X., Nephew K.P. (2019). Ovarian Cancer Stem Cells: Role in Metastasis and Opportunity for Therapeutic Targeting. Cancers.

[70] Bartakova A., Michalova K., Presl J., Vlasak P., Kostun J., Bouda J. (2018). CD44 as a cancer stem cell marker and its prognostic value in patients with ovarian carcinoma. J. Obstet. Gynaecol..

[71] Shah V., Taratula O., Garbuzenko O.B., Taratula O.R., Rodriguez-Rodriguez L., Minko T. (2013). Targeted nanomedicine for suppression of CD44 and simultaneous cell death induction in ovarian cancer: An optimal delivery of siRNA and anticancer drug. Clin. Cancer Res..

[72] Skubitz A.P., Taras E.P., Boylan K.L., Waldron N.N., Oh S., Panoskaltsis-Mortari A., Vallera D.A. (2013). Targeting CD133 in an in vivo ovarian cancer model reduces ovarian cancer progression. Gynecol. Oncol..

[73] Yang B., Yan X., Liu L., Jiang C., Hou S. (2017). Overexpression of the cancer stem cell marker CD117 predicts poor prognosis in epithelial ovarian cancer patients: Evidence from meta-analysis. Onco Targets Ther..

[74] Shaw T.J., Vanderhyden B.C. (2007). AKT mediates the pro-survival effects of KIT in ovarian cancer cells and is a determinant of sensitivity to imatinib mesylate. Gynecol. Oncol..

[75] Schilder R.J., Sill M.W., Lee R.B., Shaw T.J., Senterman M.K., Klein-Szanto A.J., Miner Z., Vanderhyden B.C. (2008). Phase II evaluation of imatinib mesylate in the treatment of recurrent or persistent epithelial ovarian or primary peritoneal carcinoma: A Gynecologic Oncology Group Study. J. Clin. Oncol..

[76] Nakamura K., Terai Y., Tanabe A., Ono Y.J., Hayashi M., Maeda K., Fujiwara S., Ashihara K., Nakamura M., Tanaka Y. (2017). CD24 expression is a marker for predicting clinical outcome and regulates the epithelial-mesenchymal transition in ovarian cancer via both the Akt and ERK pathways. Oncol. Rep..

[77] Zhang R., Shi H., Chen Z., Wu Q., Ren F., Huang H. (2011). Effects of metastasis-associated in colon cancer 1 inhibition by small hairpin RNA on ovarian carcinoma OVCAR-3 cells. J. Exp. Clin. Cancer Res..

[78] Rao Y., Ji M., Chen C., Shi H. (2013). Effect of siRNA targeting MTA1 on metastasis malignant phenotype of ovarian cancer A2780 cells. J. Huazhong Univ. Sci. Technol. Med. Sci..

[79] Huo X., Ren L., Shang L., Wang X., Wang J. (2011). Effect of WT1 antisense mRNA on the induction of apoptosis in ovarian carcinoma SKOV3 cells. Eur. J. Gynaecol. Oncol..

[80] De P., Aske J., Dey N. (2019). RAC1 Takes the Lead in Solid Tumors. Cells.

[81] Hudson L., Gillette J., Kang H., Rivera M., Wandinger-Ness A. (2018). Ovarian Tumor Microenvironment Signaling:Convergence on the Rac1 GTPase. Cancers.

[82] Lu L., Yu X. (2009). The balance of Polo-like kinase 1 in tumorigenesis. Cell Div..

[83] Zhang R., Shi H., Ren F., Liu H., Zhang M., Deng Y., Li X. (2015). Misregulation of polo-like protein kinase 1, P53 and P21WAF1 in epithelial ovarian cancer suggests poor prognosis. Oncol. Rep..

[84] Farra R., Dapas B., Grassi M., Benedetti F., Grassi G. (2019). E2F1 as a molecular drug target in ovarian cancer. Expert Opin. Ther. Targets.

[85] Oku Y., Nishiya N., Tazawa T., Kobayashi T., Umezawa N., Sugawara Y., Uehara Y. (2018). Augmentation of the therapeutic efficacy of WEE1 kinase inhibitor AZD1775 by inhibiting the YAP-E2F1-DNA damage response pathway axis. FEBS Open Bio.

[86] Ryo A., Liou Y.C., Wulf G., Nakamura M., Lee S.W., Lu K.P. (2002). PIN1 is an E2F target gene essential for Neu/Ras-induced transformation of mammary epithelial cells. Mol. Cell. Biol..

[87] Liou Y.C., Zhou X.Z., Lu K.P. (2011). Prolyl isomerase Pin1 as a molecular switch to determine the fate of phosphoproteins. Trends Biochem. Sci..

[88] Russo S.C., De S.L., Palazzolo S., Salis B., Granchi C., Minutolo F., Tuccinardi T., Fratamico R., Crotti S., D’Aronco S. (2018). Liposomal delivery of a Pin1 inhibitor complexed with cyclodextrins as new therapy for high-grade serous ovarian cancer. J. Control. Release.

[89] Russo S.C., De S.L., Poli G., Granchi C., El B.M., Ecca F., Grassi G., Grassi M., Canzonieri V., Giordano A. (2019). Virtual screening identifies a PIN1 inhibitor with possible antiovarian cancer effects. J. Cell. Physiol..

[90] Van Beijnum J.R., Petersen K., Griffioen A.W. (2009). Tumor endothelium is characterized by a matrix remodeling signature. Front. Biosci. (Sch. Ed.).

[91] Ren F., Shen J., Shi H., Hornicek F.J., Kan Q., Duan Z. (2016). Novel mechanisms and approaches to overcome multidrug resistance in the treatment of ovarian cancer. Biochim. Biophys. Acta.

[92] Trnski D., Gregoric M., Levanat S., Ozretic P., Rincic N., Vidakovic T.M., Kalafatic D., Maurac I., Oreskovic S., Sabol M. (2019). Regulation of Survivin Isoform Expression by GLI Proteins in Ovarian Cancer. Cells.

[93] Togashi K., Okada M., Yamamoto M., Suzuki S., Sanomachi T., Seino S., Yamashita H., Kitanaka C. (2018). A Small-molecule Kinase Inhibitor, CEP-1347, Inhibits Survivin Expression and Sensitizes Ovarian Cancer Stem Cells to Paclitaxel. Anticancer Res..

[94] Levy A., Alhazzani K., Dondapati P., Alaseem A., Cheema K., Thallapureddy K., Kaur P., Alobid S., Rathinavelu A. (2019). Focal Adhesion Kinase in Ovarian Cancer: A Potential Therapeutic Target for Platinum and Taxane-Resistant Tumors. Curr. Cancer Drug Targets.

[95] Yang Y., Li S., Sun Y., Zhang D., Zhao Z., Liu L. (2019). Reversing platinum resistance in ovarian cancer multicellular spheroids by targeting Bcl-2. Onco Targets Ther..

[96] (2011). Integrated genomic analyses of ovarian carcinoma. Nature.

[97] Domcke S., Sinha R., Levine D.A., Sander C., Schultz N. (2013). Evaluating cell lines as tumour models by comparison of genomic profiles. Nat. Commun..

[98] Cho K.R., Shih I. (2009). Ovarian cancer. Annu. Rev. Pathol..

[99] Clevers H. (2016). Modeling Development and Disease with Organoids. Cell.

[100] Jabs J., Zickgraf F.M., Park J., Wagner S., Jiang X., Jechow K., Kleinheinz K., Toprak U.H., Schneider M.A., Meister M. (2017). Screening drug effects in patient-derived cancer cells links organoid responses to genome alterations. Mol. Syst. Biol..

[101] Kopper O., de Witte C.J., Lohmussaar K., Valle-Inc J.E., Hami N., Kester L., Balgobind A.V., Korving J., Proost N., Begthel H. (2019). An organoid platform for ovarian cancer captures intra- and interpatient heterogeneity. Nat. Med..

[102] Maru Y., Tanaka N., Itami M., Hippo Y. (2019). Efficient use of patient-derived organoids as a preclinical model for gynecologic tumors. Gynecol. Oncol..

[103] McCloskey C.W., Rodriguez G.M., Galpin K.J.C., Vanderhyden B.C. (2018). Ovarian Cancer Immunotherapy: Preclinical Models and Emerging Therapeutics. Cancers.

[104] Bobbs A.S., Cole J.M., Cowden Dahl K.D. (2015). Emerging and Evolving Ovarian Cancer Animal Models. Cancer Growth Metastasis.

[105] Scott C.L., Becker M.A., Haluska P., Samimi G. (2013). Patient-derived xenograft models to improve targeted therapy in epithelial ovarian cancer treatment. Front. Oncol..

[106] Roby K.F., Taylor C.C., Sweetwood J.P., Cheng Y., Pace J.L., Tawfik O., Persons D.L., Smith P.G., Terranova P.F. (2000). Development of a syngeneic mouse model for events related to ovarian cancer. Carcinogenesis.

[107] McCloskey C.W., Goldberg R.L., Carter L.E., Gamwell L.F., Al-Hujaily E.M., Collins O., Macdonald E.A., Garson K., Daneshmand M., Carmona E. (2014). A new spontaneously transformed syngeneic model of high-grade serous ovarian cancer with a tumor-initiating cell population. Front. Oncol..

[108] Barba A.A., Cascone S., Caccavo D., Lamberti G., Chiarappa G., Abrami M., Grassi G., Grassi M., Tomaiuolo G., Guido S. (2017). Engineering approaches in siRNA delivery. Int. J. Pharm..

[109] Chiarappa G., Abrami M., Dapas B., Farra R., Trebez F., Musiani F., Musiani F., Grassi G., Grassi M. (2017). Mathematical Modeling of Drug Release from Natural Polysaccharides Based Matrices. Nat. Prod. Commun..

[110] Bartlett D.W., Davis M.E. (2006). Insights into the kinetics of siRNA-mediated gene silencing from live-cell and live-animal bioluminescent imaging. Nucleic Acids Res..

[111] Polyak D., Krivitsky A., Scomparin A., Eliyahu S., Kalinski H., Avkin-Nachum S., Satchi-Fainaro R. (2017). Systemic delivery of siRNA by aminated poly(α)glutamate for the treatment of solid tumors. J. Control. Release.

[112] Risnayanti C., Jang Y.S., Lee J., Ahn H.J. (2018). PLGA nanoparticles co-delivering MDR1 and BCL2 siRNA for overcoming resistance of paclitaxel and cisplatin in recurrent or advanced ovarian cancer. Sci. Rep..

[113] Hazekawa M., Nishinakagawa T., Kawakubo-Yasukochi T., Nakashima M. (2019). Glypican-3 gene silencing for ovarian cancer using siRNA-PLGA hybrid micelles in a murine peritoneal dissemination model. J. Pharmacol. Sci..

[114] Stadlmann S., Gueth U., Baumhoer D., Moch H., Terracciano L., Singer G. (2007). Glypican-3 expression in primary and recurrent ovarian carcinomas. Int. J. Gynecol. Pathol..

[115] Lou B., Beztsinna N., Mountrichas G., van den Dikkenberg J.B., Pispas S., Hennink W.E. (2017). Small nanosized poly(vinyl benzyl trimethylammonium chloride) based polyplexes for siRNA delivery. Int. J. Pharm..

[116] Leung C.S., Yeung T.L., Yip K.P., Wong K.K., Ho S.Y., Mangala L.S., Sood A.K., Lopez-Berestein G., Sheng J., Wong S.T. (2018). Cancer-associated fibroblasts regulate endothelial adhesion protein LPP to promote ovarian cancer chemoresistance. J. Clin. Investig..

[117] Kim G.H., Won J.E., Byeon Y., Kim M.G., Wi T.I., Lee J.M., Park Y.Y., Lee J.W., Kang T.H., Jung I.D. (2018). Selective delivery of PLXDC1 small interfering RNA to endothelial cells for anti-angiogenesis tumor therapy using CD44-targeted chitosan nanoparticles for epithelial ovarian cancer. Drug Deliv..

[118] Byeon Y., Lee J.W., Choi W.S., Won J.E., Kim G.H., Kim M.G., Wi T.I., Lee J.M., Kang T.H., Jung I.D. (2018). CD44-Targeting PLGA Nanoparticles Incorporating Paclitaxel and FAK siRNA Overcome Chemoresistance in Epithelial Ovarian Cancer. Cancer Res..

[119] Hong S.S., Zhang M.X., Zhang M., Yu Y., Chen J., Zhang X.Y., Xu C.J. (2018). Follicle-stimulating hormone peptide-conjugated nanoparticles for targeted shRNA delivery lead to effective gro-alpha silencing and antitumor activity against ovarian cancer. Drug Deliv..

[120] Zhang X.Y., Chen J., Zheng Y.F., Gao X.L., Kang Y., Liu J.C., Cheng M.J., Sun H., Xu C.J. (2009). Follicle-stimulating hormone peptide can facilitate paclitaxel nanoparticles to target ovarian carcinoma in vivo. Cancer Res..

[121] Yang G., Rosen D.G., Zhang Z., Bast R.C., Mills G.B., Colacino J.A., Mercado-Uribe I., Liu J. (2006). The chemokine growth-regulated oncogene 1 (Gro-1) links RAS signaling to the senescence of stromal fibroblasts and ovarian tumorigenesis. Proc. Natl. Acad. Sci. USA.

[122] Jones S.K., Douglas K., Shields A.F., Merkel O.M. (2018). Correlating quantitative tumor accumulation and gene knockdown using SPECT/CT and bioluminescence imaging within an orthotopic ovarian cancer model. Biomaterials.

[123] Lee J., Ahn H.J. (2018). PEGylated DC-Chol/DOPE cationic liposomes containing KSP siRNA as a systemic siRNA delivery Carrier for ovarian cancer therapy. Biochem. Biophys. Res. Commun..

[124] She Z.Y., Yang W.X. (2017). Molecular mechanisms of kinesin-14 motors in spindle assembly and chromosome segregation. J. Cell Sci..

[125] Fujiwaki R., Hata K., Nakayama K., Fukumoto M., Miyazaki K. (2000). Thymidylate synthase expression in epithelial ovarian cancer: Relationship with thymidine phosphorylase expression and prognosis. Oncology.

[126] Iizuka K., Jin C., Eshima K., Hong M.H., Eshima K., Fukushima M. (2018). Anticancer activity of the intraperitoneal-delivered DFP-10825, the cationic liposome-conjugated RNAi molecule targeting thymidylate synthase, on peritoneal disseminated ovarian cancer xenograft model. Drug Des. Dev. Ther..

[127] Mendes L.P., Sarisozen C., Luther E., Pan J., Torchilin V.P. (2019). Surface-engineered polyethyleneimine-modified liposomes as novel carrier of siRNA and chemotherapeutics for combination treatment of drug-resistant cancers. Drug Deliv..

[128] Blanco A., Giger-Pabst U., Solass W., Zieren J., Reymond M.A. (2013). Renal and hepatic toxicities after pressurized intraperitoneal aerosol chemotherapy (PIPAC). Ann. Surg. Oncol..

[129] Minnaert A.K., Dakwar G.R., Benito J.M., Garcia Fernandez J.M., Ceelen W., De Smedt S.C., Remaut K. (2017). High-Pressure Nebulization as Application Route for the Peritoneal Administration of siRNA Complexes. Macromol. Biosci..

[130] Dykxhoorn D.M., Palliser D., Lieberman J. (2006). The silent treatment: siRNAs as small molecule drugs. Gene Ther..

[131] Cejka D., Losert D., Wacheck V. (2006). Short interfering RNA (siRNA): Tool or therapeutic?. Clin. Sci..

[132] Bochicchio S., Dapas B., Russo I., Ciacci C., Piazza O., De Smedt S., Pottie E., Barba A.A., Grassi G. (2017). In vitro and ex vivo delivery of tailored siRNA-nanoliposomes for E2F1 silencing as a potential therapy for colorectal cancer. Int. J. Pharm..

[133] TKM 080301 for Primary or Secondary Liver Cancer. https://clinicaltrials.gov/ct2/show/record/NCT01437007?term=siRNA&cond=Ovarian+Cancer&rank=1.

[134] D’Apolito R., Tomaiuolo G., Taraballi F., Minardi S., Kirui D., Liu X., Cevenini A., Palomba R., Ferrari M., Salvatore F. (2015). Red blood cells affect the margination of microparticles in synthetic microcapillaries and intravital microcirculation as a function of their size and shape. J. Control.

